# Tiger on the prowl: Invasion history and spatio-temporal genetic structure of the Asian tiger mosquito *Aedes albopictus* (Skuse 1894) in the Indo-Pacific

**DOI:** 10.1371/journal.pntd.0005546

**Published:** 2017-04-14

**Authors:** Andrew J. Maynard, Luke Ambrose, Robert D. Cooper, Weng K. Chow, Joseph B. Davis, Mutizwa O. Muzari, Andrew F. van den Hurk, Sonja Hall-Mendelin, Jeomhee M. Hasty, Thomas R. Burkot, Michael J. Bangs, Lisa J. Reimer, Charles Butafa, Neil F. Lobo, Din Syafruddin, Yan Naung Maung Maung, Rohani Ahmad, Nigel W. Beebe

**Affiliations:** 1 University of Queensland, Brisbane, Australia; 2 Australian Army Malaria Institute, Brisbane, Australia; 3 Cairns and Hinterland Hospital and Health Service, Department of Health, Queensland Government, Cairns, Australia; 4 Forensic and Scientific Services, Department of Health, Queensland Government, Brisbane, Australia; 5 Hawaii Department of Health, Honolulu, Hawaii, United States of America; 6 Division of Parasitic Diseases and Malaria, Centers for Disease Control and Prevention, Atlanta, Georgia, United States of America; 7 Australian Institute of Tropical Health and Medicine, James Cook University, Cairns, Australia; 8 Public Health & Malaria Control Program, International SOS & PT Freeport Indonesia, Papua, Indonesia; 9 Liverpool School of Tropical Medicine, Liverpool, United Kingdom; 10 Ministry of Health, Honiara, Solomon Islands; 11 University of Notre Dame, Notre Dame, Indiana, United States of America; 12 Eijkman Institute for Molecular Biology, Jakarta, Indonesia; 13 Department of Medical Research, Yangon, Myanmar; 14 Medical Entomology Unit, Institute of Medical Research, Kuala Lumpur, Malaysia; 15 CSIRO, Dutton Park, Brisbane, Australia; University of Texas Medical Branch, UNITED STATES

## Abstract

**Background:**

Within the last century, increases in human movement and globalization of trade have facilitated the establishment of several highly invasive mosquito species in new geographic locations with concurrent major environmental, economic and health consequences. The Asian tiger mosquito, *Aedes albopictus*, is an extremely invasive and aggressive daytime-biting mosquito that is a major public health threat throughout its expanding range.

**Methodology/Principal findings:**

We used 13 nuclear microsatellite loci (on 911 individuals) and mitochondrial *COI* sequences to gain a better understanding of the historical and contemporary movements of *Ae*. *albopictus* in the Indo-Pacific region and to characterize its population structure. Approximate Bayesian computation (ABC) was employed to test competing historical routes of invasion of *Ae*. *albopictus* within the Southeast (SE) Asian/Australasian region. Our ABC results show that *Ae*. *albopictus* was most likely introduced to New Guinea via mainland Southeast Asia, before colonizing the Solomon Islands via either Papua New Guinea or SE Asia. The analysis also supported that the recent incursion into northern Australia’s Torres Strait Islands was seeded chiefly from Indonesia. For the first time documented in this invasive species, we provide evidence of a recently colonized population (the Torres Strait Islands) that has undergone rapid temporal changes in its genetic makeup, which could be the result of genetic drift or represent a secondary invasion from an unknown source.

**Conclusions/Significance:**

There appears to be high spatial genetic structure and high gene flow between some geographically distant populations. The species' genetic structure in the region tends to favour a dispersal pattern driven mostly by human movements. Importantly, this study provides a more widespread sampling distribution of the species’ native range, revealing more spatial population structure than previously shown. Additionally, we present the most probable invasion history of this species in the Australasian region using ABC analysis.

## Author summary

The Asian tiger mosquito, *Aedes albopictus*, is an aggressive mosquito that has expanded globally in the last century, chiefly due to the increase of human movements. It is a major public health concern due to its role in transmitting dengue, chikungunya and Zika viruses. Understanding how populations of *Ae*. *albopictus* are genetically related and how they have been introduced into new regions is important for controlling them and assessing their disease risk; few studies have explored this in the Indo-Pacific. In our study, we sampled a broader range of populations of *Ae*. *albopictus* in the Indo-Pacific to explore genetic patterns and to investigate the likely route of invasion through Australasia. We uncovered clear genetic groups throughout this region, but also found that some geographically distant populations are closely related, likely due to human-associated movements. We also found, that *Ae*. *albopictus* likely colonized New Guinea from mainland Southeast (SE) Asia before spreading to the Solomon Islands via either PNG or SE Asia. In contrast, the populations in Australia’s Torres Strait Islands were introduced from Indonesia. Interestingly, we found major genetic changes over time in some Torres Strait populations, less than a decade after its introduction, potentially reflective of a random reduction in genetic diversity (genetic drift) or a secondary invasion.

## Introduction

Many species of mosquitoes are amongst the most invasive pests in the world. They have a long history of human-mediated introductions [1, 2] that have resulted in the spread of major epidemics (malaria, dengue, Zika, etc.) and the establishment of invading mosquitoes as a biting nuisance. The Asian tiger mosquito, *Aedes albopictu*s (Skuse 1894) [3], is regarded as one of the most invasive mosquitoes in the world [4]. Native to tropical and subtropical Asia and multiple Western Pacific and Indian Ocean islands, *Ae*. *albopictus* now has a pan-global distribution [5–9]. Its initial movement from Southeast (SE) Asia toward the Indo-Malayan Peninsula and Indian Ocean islands may have resulted from the increase in human migration during the 17^th^ and 18^th^ centuries, with international trade (particularly the used-tire and ornamental bamboo trades) further facilitating its global spread in the 20^th^ century [10]. It is among the primary vectors of several globally expanding and medically important arthropod borne viruses (arboviruses)–particularly dengue, chikungunya, yellow fever, and Zika—while also able to transmit at least 23 other arboviruses and canine heartworm [10]. Whilst at present, the yellow fever mosquito *Aedes aegypti* (Linnaeus 1762) [11] is responsible for most of the transmission of some of these important arboviruses, the increased cold tolerance of *Ae*. *albopictus* relative to *Ae*. *aegypti* suggests that it could extend the range of many of these diseases under the right circumstances [12, 13].

Genetic techniques can provide critical information to infer the routes and sources of invasive species as well as informing on the demographic history and genetic composition of founding populations [8, 14]. For mosquito vectors, this knowledge is not only useful for inferring invasion routes in order to focus biosecurity efforts, it can also inform us of the colonizing capacity, adaptability and behaviour of invading mosquito lineages [15, 16]. However, the high dispersibility of *Ae*. *albopictus* mediated by human activities can make it challenging to detect genetic variation between populations due to high gene flow facilitated by these activities [17]. Microsatellite markers have been shown to be useful for exploring *Ae*. *albopictus* genetic patterns as they evolve rapidly and can often detect subtle population structure [8, 18, 19]. Recently, approximate Bayesian computation (ABC) analysis has proven a powerful tool in testing the probability of competing invasion scenarios. This can provide us with crucial information regarding the timing and origin of mosquito introductions, which has been used recently for *Aedes* mosquitoes [20–22].

Overall, the population genetics of *Ae*. *albopictus* through the SE Asian-Indo-Pacific region requires further exploration and samples from this region (particularly Australasia) are often lacking from global population genetic studies, despite the importance of this region for vector research [23–25]. A study by Beebe, Ambrose [18] explored part of the Indo-Australasian invasion by *Ae*. *albopictus* and provided an interesting scenario where there is high human connectivity (largely maritime) spanning both oceanic barriers and complex geographic landscapes. The current study expands on this work to include SE Asian native populations (Myanmar, Thailand, Malaysia, Singapore, Indonesia), as well as several younger populations that appear to have been introduced within the last six decades (Papua, Papua New Guinea (PNG), Solomon Islands, Fiji, Christmas Is., Cocos (Keeling) Is., Nauru, Torres Strait Islands (Australia)). Additionally, we included populations from the United States (USA) and northern Asia (only for *COI*) to see how these populations fit into a broader geographic analysis. We use previously developed nuclear microsatellite markers [18] and mitochondrial *cytochrome c oxidase subunit I* (*COI*) sequences to investigate the population genetics of the *Ae*. *albopictus* within the Indo-Pacific region. Our primary aims were to uncover the most likely historical invasion route of *Ae*. *albopictus* into the Australasian region as well as to detail the genetic connectivity and population structure of *Ae*. *albopictus* throughout this broad geographic region that we refer to as the Indo-Pacific (the aforementioned populations). While there are some records of the progressive establishment of *Ae*. *albopictus* throughout this region, the origin/s and the precise timing of introductions require testing using genetic methods under a coalescent-based approach such as ABC analysis. Our secondary aim was to further investigate the 2005 colonization of the Torres Strait Islands, Australia [26]. Many of the Torres Strait Islands have undergone intense spraying efforts since the establishment of *Ae*. *albopictus* and the region experiences monsoon-dry seasons leading to regular population bottlenecks [27, 28]. We hypothesized that the genetic changes in neutral alleles may be detectable over time in these newly invaded and small island populations.

## Materials and methods

### Ethics statement

Mosquito collections involving HLC from the Solomon Islands (S1 Table) were approved by the Medical Research Ethics Committee in compliance with Australia’s National Statement on Ethical Conduct in Human Research (project no. 2011000603). Collectors involved in HLC took anti-malarial medication and wore long-sleeved, protective clothing.

### Mosquito samples

**Fig 1:**
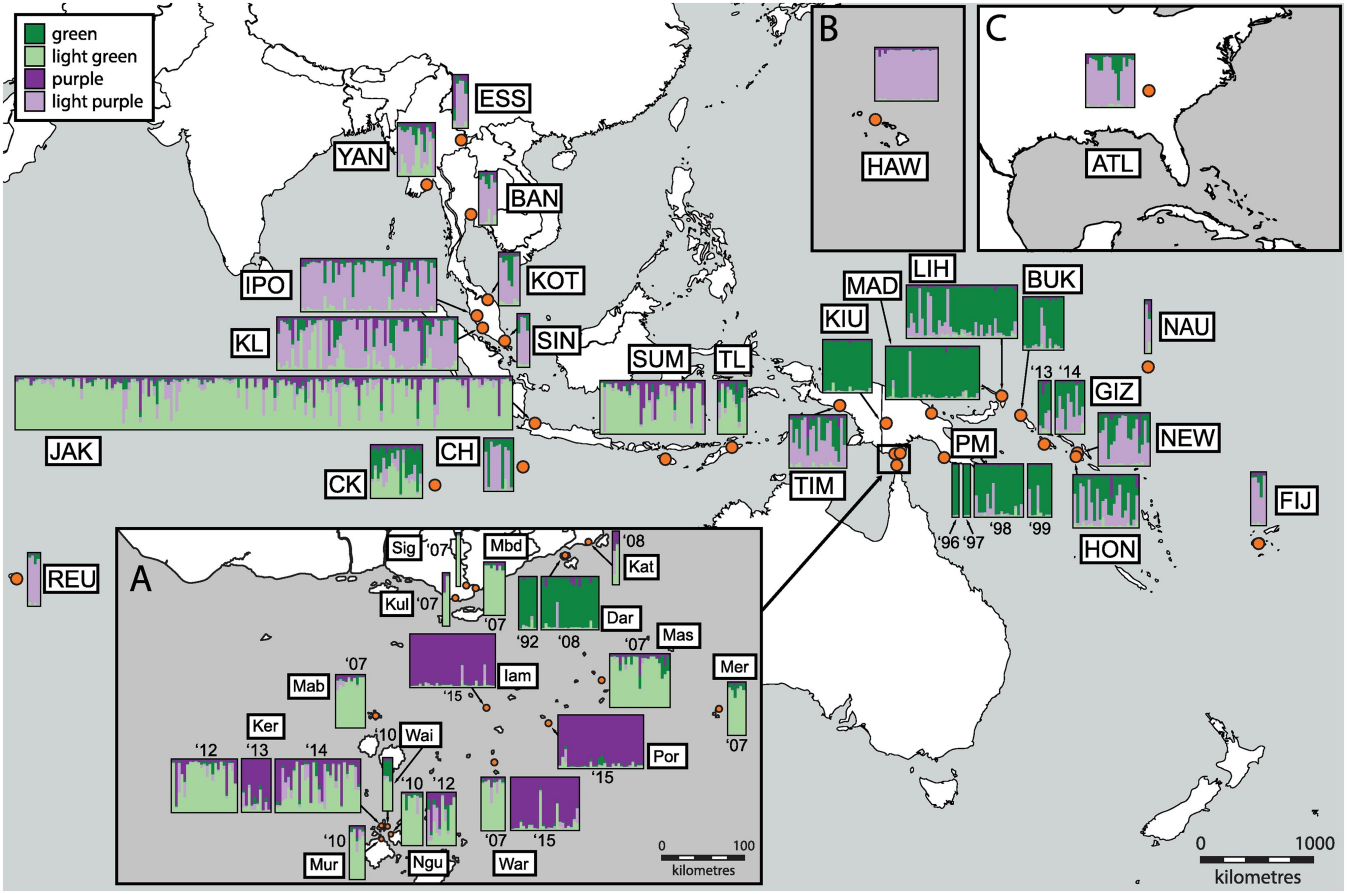
Bayesian STRUCTURE plot (K = 4) for 13 microsatellite loci for 911 samples of *Aedes albopictus* in the study region. Each vertical bar in the plots represents an individual sample, where the color of the bar indicates the probability of the individual belonging to a genetic cluster. Samples are positioned on the map corresponding to the population’s location (orange dot) and are abbreviated as in Table 1. Map insets represent the following: **A)** Torres Strait Islands and Southern Fly Region of Papua New Guinea; **B)** Hawaii; **C)** Atlanta. Insets B and C are to scale with the main map scale. The top-left color key shows the color of clusters, as referred to in the main text.

Both adult and larval samples were collected throughout Australasia, SE Asia, Indian Ocean and Pacific Ocean islands as well as in the United States (Table 1, Fig 1 (orange dots)). Samples were stored in 70% ethanol or dried (adults) over silica beads. Samples were collected using human landing captures (HLC), human baited sweep netting, egg collections and sampling of aquatic habitats for larvae and pupae (S1 Table). For identification purposes some samples were reared to adults after field collection (S1 Table). The logistics of sourcing material across multiple international borders resulted in variability in collection methods and sample sizes (Table 1, S1 Table). Adult mosquitoes were identified morphologically [29] and for larval/pupal samples using either real-time PCR assays [30] or a PCR-restriction digest [31] to differentiate from *Aedes scutellaris* (Walker 1858) [32].

**Table 1:** Sample information for *Aedes albopictus* used in the microsatellite study, where n indicates the number of individuals per population (n_total_ = 911, n_pop_ = 50). Region/description shows broader geographic regions and descriptions referred to in text. Population indicates more specific collection sites and the year of collection in brackets.

Region/description	Population (year)	n	Abbreviation	DAPC Abbreviation
Torres Strait Islands (invasion)	Masig (2007)	21	Mas '07	TS
	Mer (2007)	6	Mer '07	
	Warraber (2007)	8	War '07	
	Mabuiag (2007)	10	Mab '07	
	Waiben (2010)	3	Wai '10	
	Ngurupai (2010)	7	Ngu '10	
	Muralug (2010)	5	Mur '10	
Torres Strait Islands (post invasion)	Ngurupai (2012)	10	Ngu '12	Ngu '12
	Keriri (2012)	23	Ker '12	Ker '12
	Keriri (2013)	10	Ker '13	Ker '13
	Keriri (2014)	30	Ker '14	Ker '14
	Poruma (2015)	30	Por '15	Por '15
	Iama (2015)	30	Iam '15	Iam '15
	Warraber (2015)	24	War '15	War '15
Southern Fly Region (PNG)	Kulalai (2007)	2	Kul '07	FLY
	Mabaduan (2007)	7	Mab '07	
	Sigabaduru (2007)	1	Sig '07	
	Katatai (2008)	2	Kat '08	
Papua New Guinea	Kiunga (1992)	17	KIU	PNG
	Port Moresby (1996)	2	PM '96	
	Port Moresby (1997)	2	PM '97	
	Port Moresby (1998)	17	PM '98	
	Port Moresby (1999)	8	PM '99	
	Madang (2011)	33	MAD	
	Daru (1992)	6	DAR '92	
	Daru (2008)	20	DAR '08	
	Lihir Is. (2007)	39	LIH	
	Buka Is. (1999)	14	BUK	
Papua, Indonesia	Timika (2015)	20	TIM	TIM
Timor-Leste	Timor-Leste (2001)	10	TL	TL
Indonesia	Jakarta (2012)	177	JAK	JAK
	Sumba (2013)	37	SUM	SUM
Singapore	Singapore (2013)	4	SIN	SIN/MAL
Malaysia	Ipoh (2013)	48	IPO	
	Kota Baru (2013)	7	KOT	
	Kuala Lumpur (2015)	64	KL	
Thailand	Bangkok (2015)	6	BAN	BAN/MYA
Myanmar	Yangon (2013)	13	YAN	
	East Shan State (2013)	5	ESS	
Christmas Island	Christmas Is. (2008)	10	CH	CH
Cocos (Keeling) Islands	Direction Is. (2008)	18	CK	CK
La Réunion	La Réunion (2011)	4	REU	REU
Solomon Islands	Honiara (2013)	23	HON	SOL
	Gizo (2013)	4	GIZ '13	
	Saeragi village, Gizo (2014)	18	GIZ '14	
	New Mala (2014)	10	NEW	
Fiji	Fiji (2015)	5	FIJ	FIJ
Nauru	Nauru (2014)	2	NAU	NAU
Hawaii	Hawaii (2015)	22	HAW	HAW/ATL
USA (mainland)	Atlanta (2011)	17	ATL	

### Microsatellite analysis

DNA was salt extracted [33] and diluted at 1:10 with 1X TE buffer (Tris, EDTA). Thirteen nuclear microsatellite were used in this study. These markers were previously developed [18] and include two dinucleotide and 11 trinucleotide loci (see Beebe, Ambrose [18] for loci and primers). Some samples included from the previous study were amplified using a variation of the master mix (see Beebe, Ambrose [18]), other samples were amplified in a 15.4μl reaction that consisted of 10.8μl H_2_O, 3μl 5X Mytaq buffer (Bioline, containing 5mM dNTPs and 15mM MgCl_2_), 0.1μl 10μM M13 tagged forward primer, 0.2μl 10μM reverse primer, 0.2μl M13 tagged fluorescent dye (VIC, NED, PET or FAM; S1 Table), 0.01μl (1U) MyTaq polymerase and 1μl 1:10 DNA template. PCR cycling used the same protocol as in Beebe, Ambrose [18]. Amplification was verified by running 1μl of PCR product on a 2% agarose gel stained with either GelRed (Biotium) or MidoriGreen (Bulldog Bio). Samples that amplified successfully were sent to Macrogen Inc. (Republic of Korea) for genotyping.

GeneMarker v.2.4.2 (SoftGenetics LLC [34]) was used to score alleles for each locus manually after passing the data through the standardization run wizard using the default animal fragment setting. Random selections of genotyped plates were rescored by a second person to assess consistency in scoring. In addition to the data collected in this study, we included microsatellite scores from samples in Beebe, Ambrose [18]. During the present study, it was uncovered that the Beebe, Ambrose [18] study used (in some cases) inconsistent fluorescent dyes for a given locus, which caused a dye-shift [35] resulting in inconsistently scored alleles. We regenotyped a random subset of individuals from each of the populations used in the study by Beebe, Ambrose [18] to ensure consistency with data collected from this study. The predictability of this dye-shift (based on the dyes used previously) enabled shifting of the allele scores from Beebe, Ambrose [18] for use in this study. Samples with fewer than nine scored loci of 13 total were removed before further study as we considered these poor quality samples; thus leaving 911 samples for final analyses (20% of samples were from Beebe, Ambrose [18]; Table 1). With the remaining dataset, missing values were replaced based on population allele frequencies using GenoDive v. 2.0b27 [36]–based on preliminary analyses this did not significantly alter population structure and relationships between populations. Missing values were not replaced for the STRUCTURE analysis, calculation of HWE and for checking the presence of null alleles.

Scored allele frequencies were checked for the presence of null alleles using MICRO-CHECKER [37] and for Hardy-Weinberg equilibrium using GenAlEx v.6.5 [38, 39]. Additionally, we calculated fixation index (F), allelic richness (Na), number of effective alleles (Ne) and the observed (Ho) and expected (He; unbiased estimate: uHe) values of heterozygosity using GenAlEx v.6.5. Pairwise population indices of genetic variation for Jost’s D, G”_ST_ and F_ST_ were also calculated between populations in GenAlEx v.6.5 (S2 Table). We used 9,999 permutations and an analysis of molecular variance (AMOVA) to assess significance. A Mantel test was also performed in GenAlEx v.6.5 on geographic and genetic distance (pairwise phiPT) using 9,999 permutation [38, 39].

Population structure was investigated using the Bayesian program STRUCTURE v.2.3.4 [40] to infer the most probable number of population clusters (K). Based on our preliminary runs (S1 File, Supplementary Methods: Preliminary STRUCTURE), final analyses were run at both a lower (K = 4) and upper (K = 9) K value. For both K values we used a burn-in of 100,000 and runtime of 2,000,000 generations per iteration (20 iterations). For K = 9, cluster membership probabilities were somewhat inconsistent across runs due to multimodality; 20 iterations helped to account for this [41]. We assessed whether the burn-in period was adequate by reviewing summary statistics in STRUCTURE [41]. CLUMPP v.1.1.2 [42] was used to compile data from the 20 iterations for the independent values of K using the Greedy algorithm with 1,000 replicates. Final graphs were formed in DISTRUCT v.1.1 [43].

Discriminant analysis of principal components (DAPC) and correspondence analysis (CA) was used to further assess population structure. DAPC was implemented in R Studio v.3.2.2 (RStudio Team 2015 [44]) using the adegenet 1.4–1 package [45, 46] using the whole microsatellite dataset, where group membership was defined by the populations outlined in Table 1 (see DAPC abbreviation). These populations were more broadly defined and differed slightly from those used in STRUCTURE, to allow for easier interpretation of the results presented here. Specifically, the Torres Strait populations were split into groups based on their genetic relationship to one another and geographic/temporal information to reduce clutter in plots (Table 1; DAPC abbreviation). Only populations that were genetically similar were grouped together, which was confirmed using STRUCTURE, DAPC, CA and pairwise tests for genetic distance (F_ST_, G”_ST_, Jost’s D) on the full dataset and subsets. Final DAPC analyses were performed on both a full dataset (n_ind_ = 911, n_pop_ = 23; including all populations) and a reduced dataset (n_ind_ = 458, n_pop_ = 18; excluding Jakarta, Sumba, Timor-Leste, the Torres Strait Islands and Southern Fly Region) to help discriminate genetically similar populations.

In adegenet, cross-validation was performed on each of our DAPCs independently, using a training dataset of 90% and a validation set of 10%, using 100 replicates. The number of PCs (n.pca) associated with the lowest root mean squared error (RMSE) was used as this was considered optimum [47]. Cross-validation suggested retaining 60 PCs for the full dataset and 40 PCs (n.pc) for the reduced dataset. We used five discriminant functions (n.da) for each of the analyses, but only the first three are plotted and discussed here as they explained the majority of variance (see Results). A correspondence analysis (CA) was also implemented in adegenet on the full dataset to investigate general trends and to complement DAPC, as visualization of the data is simplified in CA because within-population genetic diversity is not displayed.

The Garza-Williamson index (M-ratio) was calculated for populations using our microsatellite dataset in Arlequin v.3.5.2.2 [48]. The M-ratio was used to investigate the demographic history of populations and to test for recent bottleneck events; wherein an index statistic closer to 1 suggests the population is in a stationary state whereas very low values suggests a population has gone through a genetic bottleneck in the past (with a critical value of 0.68 indicating a bottleneck) [49, 50]. A Wilcoxon test for heterozygosity excess was also conducted on populations to detect bottlenecks using BOTTLENECK v.1.2.02 [51]. We used a two-phase model (TPM) of mutation with 10% infinite allele model and a 90% single step mutation model with 15% variance for 1000 iterations. A Wilcoxon signed rank test (two-tailed) was used to calculate significance (P < 0.05).

### Invasion history—ABC

**Fig 2:**
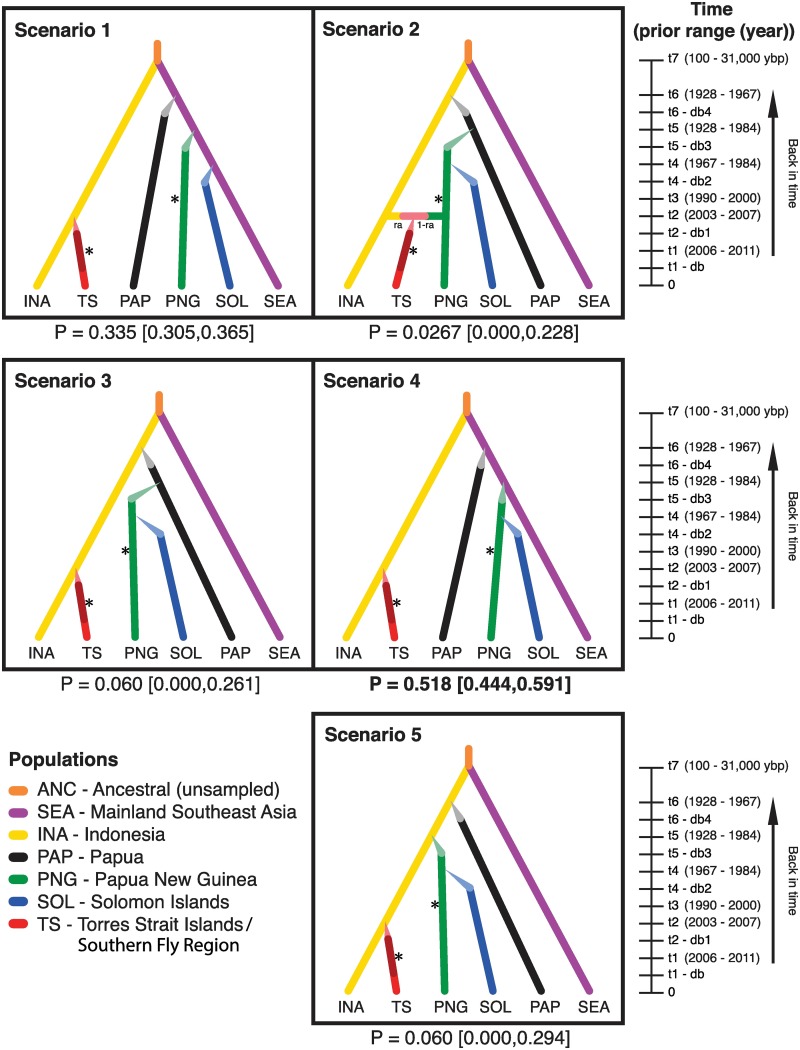
Invasion scenarios of *Aedes albopictus* in Australasia tested using approximate Bayesian computation (ABC). One unsampled and six sampled populations were modelled, shown as colored lines in five different invasion scenarios. Time events (t1-t7) are not to scale, but their prior distributions are displayed as the year (except t7 which is shown in years before present (ybp, present = 2015)). Changes in effective population size (N_e_) are represented as differently shaded lines, where db-db4 represent the duration; narrowing lines represent population bottlenecks that were given lower N_e_ priors ranges; rate of admixture (ra) is also shown. All populations have samples at time = 0 (i.e. 2015) and asterisks represent additional temporal sampling of populations (i.e. for TS and PNG). The posterior probabilities of all scenarios are shown with 95% confidence intervals in square brackets; Scenario 4 was the best-fit scenario. See S3 Table for further details and posterior distributions.

We tested the invasion history of *Ae*. *albopictus* in part of the study region (SE Asia/Australasia)—populations from the Indian Ocean, Fiji, Nauru and United States were not included due to insufficient sampling of these regions. Both *COI* sequences and the thirteen microsatellites loci were analysed together using ABC analysis in DIYABC v.2.1.0 [52]. Due to the complexity of modelling each population separately in this region, we simplified our invasion scenarios by randomly subsampling individuals from distinct geographic and genetic groups (defined using our other analyses). These representative populations included: mainland SE Asia, Indonesia, Papua, PNG, the Solomon Islands and the Torres Strait Islands/Southern Fly Region. For both the PNG and the Torres Strait/Southern Fly Region populations, we included temporal sampling in our scenarios (asterisks, Fig 2). Each of these representative populations/sampling events was made up of 30 individuals except Papua which used all 20 samples from the only sampled population, Timika. In addition, we included an unsampled ancestral population (ANC, Fig 2) in our model that split into mainland SE Asian and Indonesian populations.

Preliminary runs were carried out in accordance with Bertorelle, Benazzo [53] in order to optimise summary statistics, prior estimates and the scenarios tested. Final runs compared five invasion scenarios; the final summary statistics, prior estimates and parameter conditions used are outlined (S3 Table). Each scenario represents a plausible invasion route into the Australasian region. These were constructed using historical records regarding the timing and suspected sources of the different invasions [26, 54–58]. Priors were sampled from a wide range of distributions based on these records—less certain time priors were given a wider distribution and standard deviation whereas more likely priors were assigned narrower estimates. The upper time bound for the divergence of mainland SE Asia and Indonesia from a common ancestor was based on Porretta, Mastrantonio [59] while the lower bounds allowed for the possibility of a more recent split associated with human migration [10] (S3 Table). Because DIYABC measures time in terms of the number of generations, we assumed 10 generations per year for *Ae*. *albopictus* (which typically ranges from 5–17 generations in the tropics).

Estimates of effective population size (N_e_) ranged from 10 to 1,000,000 individuals (uniform distribution) [20–22] depending on if a population was modelled as going through a change in N_e_. For instance, each of the recently introduced populations was modelled to allow for a founder effect after its introduction using lower N_e_ ranges (Fig 2, S3 Table). We additionally allowed for a change in N_e_ in the Torres Strait Islands/Fly Region population due to the drastic temporal changes we observed in our other analyses—this would allow us to make a relative comparison of N_e_ in order to see if the population had undergone any change in N_e_ (indicative of bottleneck/expansion events) (Fig 2, S3 Table). For *COI*, we used the HKY mutation model [60] and sampled from a uniform distribution with mutation rates ranging between 7x10^-10^–1x10^-7^. For microsatellites, both di- and tri- nucleotide repeats were modelled separately due to the possibility of different repeat lengths having different mutation rates [61]. Both microsatellites used the default generalized stepwise mutation model and were assigned a loguniform distribution with mean mutation rates between 1x10^-6^–1x10^-3^ (S3 Table). All mutation rates were based on standard ranges for *COI* [62] and for Dipteran microsatellites [20–22, 63]. We simulated 15,000,000 datasets and each of the five scenarios was given a uniform probability. The performance of the ABC approach was assessed using multiple methods in DIYABC (S1 File, Supplementary Methods: Performance of DIYABC).

### *COI* analysis

The mitochondrial protein-coding gene *COI* was amplified using custom designed primers (albCOIF 5’-TTTCAACAAATCATAAAGATATTGG-3’ and albCOIR 5’- TAAACTTCTGGATGACCAAAAAATCA-3’) for 259 random individuals across different populations. Each 25.3μL reaction consisted of 19μl H_2_O, 5μl 5X Mytaq buffer (Bioline, with pre-optimized concentrations of dNTPs and MgCl_2_), 0.1μl 100μM forward primer, 0.1μl 100μM reverse primer, 0.1μl MyTaq polymerase and 1μl 1:10 DNA template. PCR used an initial denature of 94°C for 3 min, 35 cycles of denaturation at 95°C for 30 sec, primer annealing at 45°C for 40 sec, and primer extension at 72°C for 30 sec. Final elongation lasted 5 min at 72°C prior to cooling to 4°C. Amplification was confirmed using gel electrophoresis (as described previously) and PCR products were purified by adding 2μl per sample of a mixture containing equal amounts of Exonuclease I and Antarctic Phosphatase (New England Biolabs, Australia) before incubation at 37°C for 20 min and denaturation at 80°C for 10 min. Samples were sequenced by Macrogen Inc. (Republic of Korea) using Sanger sequencing. Additional *COI* sequences of *Ae*. *albopictus* were obtained from other studies and from Genbank (1044 sequences total, 259 produced in this study, S4 Table).

Sequences were edited and aligned in Geneious v.9.0.4 (http://www.geneious.com, Kearse, Moir [64]) using the MAFFT alignment. The final alignment was trimmed to 445bp to incorporate the large number of *COI* sequences available from Genbank which were smaller than the ~700bp region sequenced in this study. All sequences were checked for stop codons in Geneious v.9.0.4. TCS haplotype networks [65] were constructed using 1,000 iterations in PopArt v.1.7 (http://popart.otago.ac.nz). In addition, we calculated Tajima’s D for populations with temporal data in PopArt v.1.7 to determine whether sequences were evolving randomly or non-randomly. Haplotype and nucleotide diversity was calculated using DnaSP v.5.10.1 [66].

### Accession numbers

All new *COI* sequences generated in this study are available on Genbank: KY907195—KY907453.

## Results

### Microsatellite genetic diversity and population structure

Allelic richness for microsatellites was highest in native populations of *Ae*. *albopictu*s from Myanmar, Thailand, Malaysia, but also high in recently invaded areas such as some of the Torres Strait Islands and in PNG and the Solomon Islands (See Na in S5 Table). A Mantel test on the whole dataset showed a significant (P = 0.0001) positive, but weak, correlation (R^2^ = 0.02) between genetic (phiPT) and geographic distance (y = 0.0002*x* + 28.1). Pairwise estimates of F_ST_, Jost’s D and G”_ST_ all revealed similar results to each other and recovered mostly significant relationships between populations; here we discuss gene flow and genetic distance in regards to F_ST_ estimates but the other measures are shown in S2A–S2C Table. Lowest F_ST_ values were apparent between populations belonging to the same geographical region (for definition of regions see Table 1, Region/description), especially within mainland SE Asia (F_ST_ = 0.011–0.103) (S2A Table). However, some comparisons between regions separated by vast geographical distances also showed low F_ST_ scores, such as populations from the Solomon Islands with populations from mainland SE Asia (F_ST_ = 0.050–0.114) (S2A Table). The relationships between populations were mostly consistent with the results obtained in STRUCTURE and multivariate analyses, which are described in detail below.

Within the study region, four to nine clusters were supported by the Evanno ΔK and log likelihood methods for inferring K. While K = 4 (Fig 1) represents the simplest summary of the genetic structure of *Ae*. *albopictus* in the region, we detected substantial substructure within these four main clusters which are apparent at K = 9 (S1 Fig). We discuss the data in the context of both values of K to avoid underestimating the degree of population structure within the study region.

At K = 4, clusters mostly pertained to distinct but broad geographic boundaries although many populations and individuals show signs of admixture, despite the large geographic distances (Fig 1). The mainland SE Asian populations of Myanmar, Thailand, Malaysia and Singapore cluster with the USA (Hawaii and Atlanta; Fig 1B and 1C), La Réunion, as well as Fiji and Nauru (light purple; Fig 1). The second cluster (light green) contains populations from Indonesia (Jakarta and Sumba), Timor-Leste, the Southern Fly Region of PNG (Fig 1A) and several islands of the Torres Strait (especially collections following the first detection of *Ae*. *albopictus* in the straits in 2005 (collections between 2006–2014)) (Fig 1A). An additional cluster (purple) is prominent within the Torres Strait region (Fig 1A) and represents populations on the islands collected more recently (2013–2015), suggesting temporal shifts in population structure have occurred on some islands (see Fig 1A: Ker, War). The fourth cluster (green) is composed of historically-established PNG populations, but note that some of these populations contain admixture with the SE Asian cluster (Fig 1). The island of Daru (Fig 1A: Dar), which is less than 5km from the Southern Fly Region incursion populations (light green cluster; Fig 1A; Sig, Kul, Mbd, Kat), is distinct and clusters with the historically established PNG populations. Timika and the Solomon Islands show genetic affinity to both PNG and SE Asian clusters (Fig 1). Indian Ocean islands (Fig 1: CK and CH) appear differentiated from each other and contain a notable degree of admixture, but Christmas Is. is more similar to SE Asia whereas Cocos (Keeling) Islands appear as an admixed population made up of the PNG and Indonesian clusters. When K = 9, the same broad population patterns are observed but some populations/regions become more distinct, including the USA and Hawaii, Solomon Islands, Timika, the Cocos (Keeling) Islands and Sumba/Torres Strait Island populations (S1 Fig). Relationships for this K value are described in S1 File, Supplementary Results, STRUCTURE (K = 9).

For multivariate analyses, the DAPC on the full dataset (n.pc = 60, n.da = 5) explained 89% of variance, whereas the reduced dataset (n.pc = 40, n.da = 5) explained 79.3% of the variance in the data. Eigenvalues for these first three PCs are 206.37, 110.78 and 63.21 for the full dataset (S2A Fig) and 99.6, 69.2 and 44.99 for the reduced dataset (S3 Fig); these values correspond to the ratio of between-group over within-group variance for each discriminant function. For the correspondence analysis (CA) we plotted the first three eigenvalues (0.19, 0.15, 0.09), which indicate the proportion of variance explained by the first three PCs (S2B Fig).

DAPC and CA results of the full dataset (S2 Fig) showed similar population differentiation as observed in STRUCTURE at K = 4. Due to the large number of populations, we describe population structure based on the broad clustering—populations are color coded with geographically close populations being more similar in color. Four major clusters of populations are noticeable when the first three PCs are plotted against each other (C1-C4, S2A Fig). However, there is considerable overlap between these clusters, particularly with C4 overlapping C2 and C3, suggesting that these individuals and populations are genetically similar and show signs of admixture (S2A Fig). Cluster 1 (C1) represents recent (2012–2015) collections from Torres Strait Islands and is the most distinct from the other clusters. It is most closely related to C2, which contains earlier collections (2007–2014) from the Torres Strait Islands and populations from the Southern Fly Region, Sumba, Timor-Leste and Jakarta (S2A Fig). The relationships uncovered in the DAPC of the full dataset were recovered in the CA and are more easily visualized, where the first three principal components (PCs) of the CA are plotted in three-dimensions (S2B Fig); however, note that the large amount of within-population variation (as displayed in the DAPC plots) is not shown.

Because of the overlap of C3 and C4 clusters, we separately analysed these clusters by DAPC (referred to in the Materials & Methods as the reduced dataset) that excluded populations from the Torres Strait Islands, Jakarta, Sumba, Timor-Leste and the Southern Fly Region (S3A and S3B Fig) to explore substructure within these clusters (i.e. C3 & C4 in S2A Fig). Populations from PNG (Kiunga, Madang, Port Moresby and Daru) were similar to each other but distinct from the other populations (S3 Fig). The offshore PNG populations (Lihir Is. and Buka Is.) were somewhat differentiated from mainland PNG populations, although Buka Is. shares some overlap with both Port Moresby and Lihir Is. (S3 Fig). The Solomon Islands also appears similar to Lihir Is. and Buka Is. populations, but is somewhat distinct (S3 Fig). Mainland SE Asian populations appear genetically similar and tend to exhibit the most genetic overlap with other populations (S3 Fig). Nauru and Fiji are most similar to mainland SE Asian populations. In contrast, both USA populations (Hawaii and Atlanta) as well as La Réunion appear well differentiated from mainland SE Asian populations. Cocos (Keeling) Island and Timika share some overlap with each other, whereas Christmas Island shares overlap with both PNG and mainland SE Asia populations (S3 Fig).

### Invasion history—ABC

The scenario with the highest posterior probability using the logistic approach was scenario 4 (P = 0.52 [95% CI: 0.44, 0.59], Fig 2). In this scenario, *Ae*. *albopictus* colonized Papua and PNG in two separate events from mainland SE Asia, established in the Solomon Islands via PNG and more recently colonized the Torres Strait Islands/Southern Fly Region via Indonesia (Fig 2). The timing of each introduction event is shown in S3 Table and corresponds with historical records for the introduction of *Ae*. *albopictus* in the tested populations, although 95% confidence intervals (CI) suggest that some introduction dates could have been earlier than first observed (see Discussion). None of the other scenarios showed overlapping 95% CI with Scenario 4 (Fig 2), however, we detected moderate levels of type I (0.45; probability that scenario 4 is rejected given that it is the ‘true’ scenario) and type II error (0.48; probability of deciding scenario 4 is the ‘true’ scenario when it is not) that suggest Scenario 1 (P = 0.34 [95% CI: 0.31, 0.37]) could provide a plausible alternative invasion scenario for our data (S3 Table). Scenario 1 is identical to scenario 4, except that the Solomon Islands is modelled as originating from mainland SE Asia, rather than from PNG (Fig 2). Consequently, we discuss the Solomon Islands invasion based on both alternative origins and posterior estimates calculated under the combined scenarios are presented (in Discussion), although show no significant difference from scenario 4 alone (S3 Table). A preliminary analysis showed low support (P = 0.001 [95% CI: 0.00, 0.12]) for a scenario where the Solomon Islands introduction was modelled as an admixture event between mainland SE Asia and PNG compared to the five scenarios compared in our final analyses (but using slightly different N_e_ prior ranges for all founders (10–10,000)).

Each introduced population showed no relative change in N_e_ due to large 95% CIs of posterior distributions, although median N_e_ values were smaller for founding events (S3 Table). Likewise, the duration of the modelled bottleneck had large 95% CIs, but median values generally ranged from 16–30 generations (S3 Table). Overall, the Torres Strait Islands/Southern Fly Region population showed a stable N_e_ since its introduction (due to overlapping 95% CIs), although there was a gradual increase in median N_e_ over time, potentially suggesting growth of the population as a whole. (S3 Table). Our assessment of the performance of our ABC analysis was supported as fitting our observed data well (S4 and S5 Figs).

### *COI* haplotype networks and diversity

**Fig 3:**
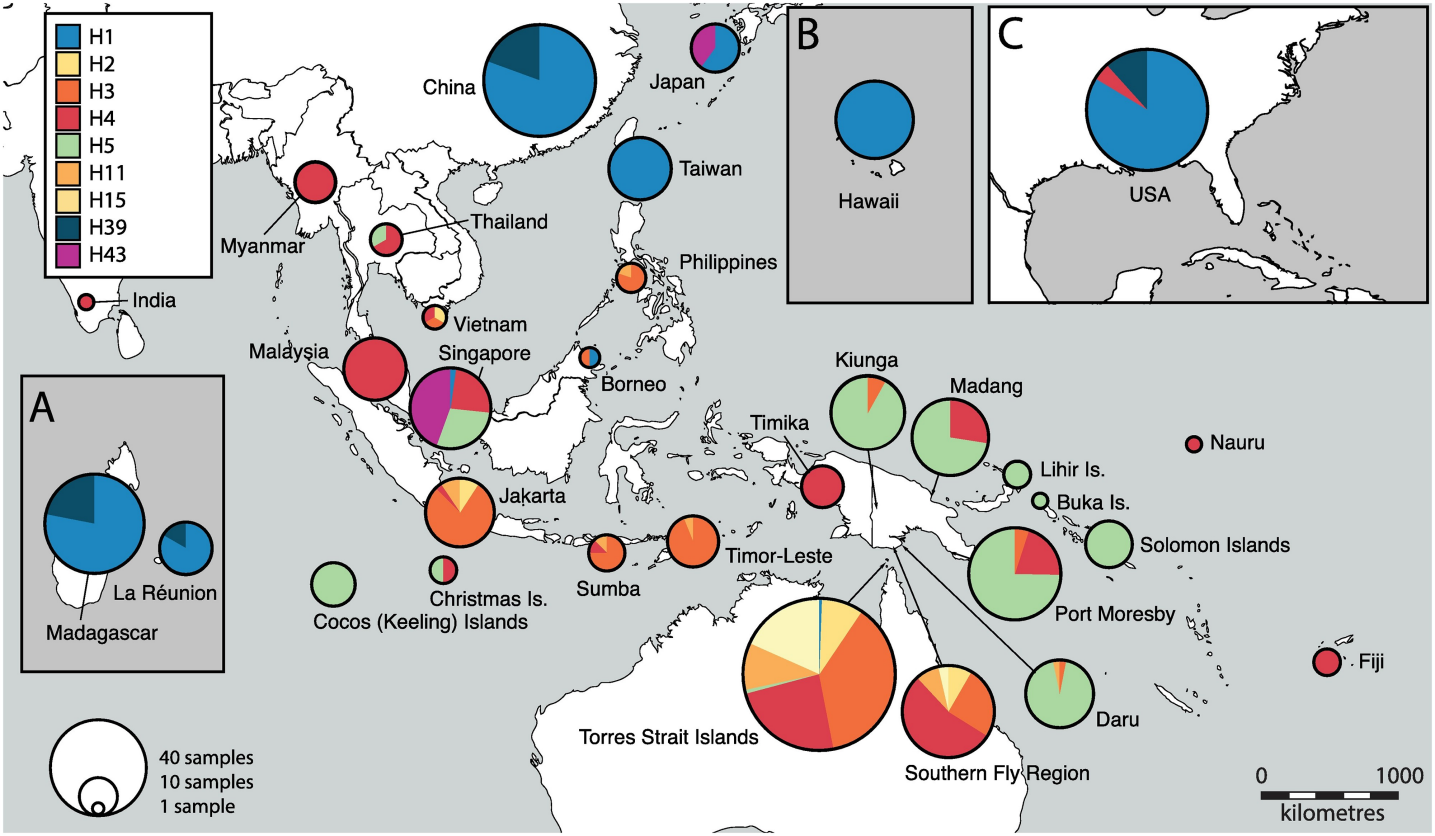
Major mitochondrial *COI* haplotypes for *Aedes albopictus* in the Indo-Pacific, Asian and USA region, representing 92% of the 1044 individuals analyzed. Displayed are the nine most prevalent *COI* haplotypes (of 52 in total) using data from ours and other studies, where each haplotype is represented as a different color and the size of the circle represents the number of individuals from a given region (which is plotted on the map). Note, the placement of circles does not correspond to the exact location of haplotypes, but represents the general region they are from; refer to S6 Table for the exact location of haplotypes and for additional haplotypes found in the region. Insets show distant regions, but are to scale with the main map: A) Madagascar and La Réunion; B) Hawaii; C) USA.

A total of 52 *COI* haplotypes were identified from the 1044 individuals used for generating the TCS haplotype network, with 92% of individuals belonging to nine main haplotypes (H1-5, 11, 15, 39, 43) (Fig 3, Table 2, S6 Fig). The distribution of these haplotypes by specific population is shown in S6 Table. All new sequences generated from this study are available on Genbank (Accession no. KY907195—KY907453; see S4 Table for accession numbers of sequences from other studies). The *COI* haplotype network is less informative in regards to population structure than the microsatellite data, although it does highlight broader geographic relationships that are somewhat consistent with the microsatellite results. Of the nine main haplotypes, H1 has the most individuals, mostly from eastern Asia (China, Taiwan and Japan), USA (mainland USA and Hawaii), Madagascar and La Réunion (Fig 3, Table 2). Similarly, H39 is distributed in a similar temperate/subtropical region. Haplotype 3 consists primarily of individuals from the Torres Strait Islands, Fly Region, Indonesia, Timor-Leste, PNG and the Philippines. However, the majority of PNG sequences belonged to H5, which also includes individuals from the Solomon Islands, Indian Ocean islands (CK and CH), Singapore and Thailand. Another major haplotype, H4, includes the most diverse range of populations (in terms of geographic spread), although it mostly consists of mainland SE Asian populations and populations from the tropics. Of the additional *COI* haplotypes, many are exclusive or shared amongst close geographic regions (Fig 3, Table 2, S6 Table, S6 Fig), although others show no apparent geographic pattern.

**Table 2:** Haplotype distribution of mitochondrial *COI* sequences for 1044 individuals of *Aedes albopictus* by broad population region. See S6 Table for a more specific summary of *COI* haplotypes by population.

Region	Haplotypes																		
	H1	H2	H3	H4	H5	H6	H10	H11	H13	H15	H23	H25	H30	H39	H40	H42	H43	H49	Exclusive Haplotypes
Torres Strait Islands	1	15	62	40	1			17	4	30									H16, H17, H19, H24
Fly Region		5	15	32				5	1	2									
Papua New Guinea			7	23	141	1	1	1											H9, H12, H29, H35
Solomon Islands					14		1												H33, H34
Fiji				4															H14
Nauru				1															
Timika				11															
Timor-Leste			16					1											
Sumba			6	1				1			1								H36, H38
Jakarta		3	25	1				3			1								H20, H21, H22
Singapore	1			11	13							1					20		H37
Malaysia (includes Borneo)	1		1	26								4	2						H7, H26, H27, H28, H31
India				1															H51
Myanmar				10									2						H32
Thailand				4	2	1													
Philippines			4					1											
Vietnam		1	1	1															
Cambodia																			H47
China	71													17	1	1			H41
Taiwan	26														3	1			
Japan	9																6		
Hawaii	40																	1	H44, H45, H46
USA (mainland)	88			5										12	1			4	H18, H48, H52
Christmas Island				2	2														H8
Cocos (Keeling) Island					12														
La Réunion	15													3					
Madagascar	54													15					H50
**TOTAL (per haplotype)**	306	24	137	173	185	2	2	29	5	32	2	5	4	47	5	2	26	5	-

Haplotype diversity (Hd) for the total dataset was high (Hd = 0.83) as was nucleotide diversity (π = 0.0037); however, population measures of Hd and π varied considerably (Table 3). Neutrality tests (Tajima’s D) on all populations with temporal data were not significant (S7 Table).

**Table 3:** Estimates of genetic diversity for the mtDNA *COI* region for populations of *Aedes albopictus* in the study. The range of collection years is shown. Number of individuals sequenced (n), number of haplotypes (n_H_), haplotype diversity (Hd) and nucleotide diversity (π) are displayed along with the total summary for all populations.

Region	Year	n	n_H_	Hd	π
Torres Strait Islands	2004–2015	175	12	0.780	0.0040
Fly Region	2007	60	6	0.648	0.0034
Papua New Guinea	1992–2011	180	10	0.370	0.0014
Solomon Islands	2013–2014	26	4	0.582	0.0028
Fiji	2015	5	2	0.4	0.0009
Nauru	2014	1	1	N/A	N/A
Timika, Papua	2015	11	1	0	0
Timor-Leste	2001	17	2	0.118	0.0003
Sumba	2013	11	6	0.727	0.0026
Jakarta	2011–2013	36	8	0.514	0.0016
Singapore	2011–2013	47	6	0.701	0.0034
Malaysia	2013	41	10	0.591	0.0020
India	2012–2014	2	2	1	0.0023
Myanmar	2013	13	3	0.410	0.0010
Thailand	2000–2015	7	3	0.666	0.0017
Vietnam	2000–2004	3	3	1	0.0045
Cambodia	2001	1	1	N/A	N/A
Philippines	2016	5	2	0.4	0.0009
China	2011	91	5	0.359	0.0009
Taiwan	2011	30	3	0.246	0.0006
Japan	2011	15	2	0.514	0.0012
Hawaii	1971–2015	45	5	0.282	0.0009
USA (mainland)	2011	129	7	0.390	0.0010
Christmas Island	2008	5	3	0.8	0.0023
Cocos (Keeling) Island	2008	12	1	0	0
La Réunion	2000–2011	18	2	0.294	0.0007
Madagascar	2007–2009	70	3	0.364	0.0008
All populations		1044	52	0.834	0.0037

### Bottleneck tests

Past genetic bottleneck events were not consistently indicated using both Wilcoxon tests for heterozygosity excess and M-ratio, with the exception of the 2010 population from Waiben of the Torres Strait (M = 0.68, P = 0.032 (two-tailed Wilcoxon signed rank test for heterozygosity excess) (S8 Table). However, multiple populations showed signs of a bottleneck using a single method. The M-ratio indicated a bottleneck for some populations of the Torres Strait Islands (Mabuiag, Waiben, Ngurupai, Poruma, Iama), Port Moresby, Timika and Gizo, whereas the Wilcoxon test was significant for Waiben, Madang, Jakarta, Yangon and the Cocos (Keeling) Islands (S8 Table).

## Discussion

The population structure and genetic connectivity of *Ae*. *albopictus* within the Indo-Pacific region has been limited to a few studies that only examined regional structure or had restricted sampling within the species’ range. In addition, the genetic characteristics of some populations examined in this study have been unexplored (e.g. Solomon Islands, many Indian and Pacific Ocean Islands, Papua-Indonesia). Using multiple lines of evidence (and both microsatellite and mitochondrial markers), we show high spatial genetic structure throughout this region. We used coalescent ABC analysis to test for the first time the likely invasion route of *Ae*. *albopictus* into the Australasian region and uncovered that the species likely invaded New Guinea from mainland SE Asia and the Solomon Islands via either PNG or SE Asia. We also show the recent invasion of *Ae*. *albopictus* into northern Australia’s Torres Strait region and Southern Fly Region of PNG likely originated from Indonesia, as previously suspected [18]. Furthermore, we provide evidence of rapid temporal shifts in population structure occurring less than a decade after the Asian tiger mosquito’s introduction into Australia’s Torres Strait Islands in 2005 [26]. In contrast, historically-introduced and native populations of *Ae*. *albopictus* showed less spatial population structure at a regional level, despite large geographic distances and international boundaries between some of these populations. Importantly, this study provides a widespread sampling distribution of the species’ native range and revealed more spatial population structure than previously shown, as well as evidence for rapid temporal genetic change in newly established populations in the Torres Strait Islands.

### Population structure in the Indo-Pacific

Multiple population studies have attempted to capture the amount of genetic structure throughout the species’ native range. Allozyme studies have shown that Indonesian and Japanese populations of *Ae*. *albopictus* are likely distinct [67] and that SE Asia (Borneo, peninsula Malaysia) and southern Asian populations (India, Sri Lanka) can both be differentiated from northern Asian populations (China, Japan) [68]. While no studies have conducted a comprehensive analysis of the species’ full native range [8], the genetic differentiation of native Asian populations of *Ae*. *albopictus* may confer to both a north-south (Korea to Indonesia) and east-west (Japan to India) pattern of genetic differentiation. Our results partly support this pattern, with evidence for genetic differentiation separating northern Asia (*COI* data only, no microsatellite data available), SE Asia and Indonesia (both *COI* and microsatellite data). Within mainland SE Asia, our data revealed little to no population structure despite high genetic diversity and *COI* haplotype diversity, supporting the findings of other studies [59, 69, 70]. Using climatic modelling and two mitochondrial markers, Porretta, Mastrantonio [59] suggested that the low genetic structure across this mainland SE Asian region could be explained by the demographic growth between interconnected populations of *Ae*. *albopictus* preceding the last glacial maximum (LGM, occurring ~21,000 ybp), with the species’ ecological flexibility facilitating its success in the ecologically diverse Sundaland (exposed SE Asian landmass) during this period. Whilst their study lacked sampling from Indonesia, their data suggested climatically suitable habitat for *Ae*. *albopictus* existed across the southern range of Sundaland which later formed the Indonesian islands after a rise in sea levels. They hypothesized that the emergence of Sundaland during the LGM could have facilitated population connectivity across Indonesia and mainland Asia. In contrast, we found clear genetic differentiation between the Indonesian archipelago and mainland SE Asia with both our microsatellite and *COI* data, which could be explained by fragmentation and subsequent differentiation of *Ae*. *albopictus* populations following a rise in sea levels or potentially driven by human migration from the region [10]. Our ABC analysis supports a more historical split ~9,040 ybp (95% CI = 1,790–27,600 ybp) but further investigation with extensive native population sampling would be needed to clarify dating. The genetic homogeneity and high gene flow (F_ST_ = 0.020–0.103) in our microsatellite data across mainland SE Asia (including Malaysia, Singapore, Myanmar and Thailand) could also be explained by human-mediated gene flow associated with transportation infrastructure (land, air and sea); aircraft and road networks in particular may be major drivers in the connectivity of *Ae*. *albopictus* in this region given the inland location of many of these populations [71–73]. For our full dataset, the relationship between genetic and geographic distance was significant, however, the correlation was weak (R^2^ = 0.02), highlighting the potential extant that human movements have had on *Ae*. *albopictus* population structure; although it does also highlight a minor trend of isolation by distance in our study region.

A recent worldwide study that examined the mitogenome diversity of *Ae*. *albopictus* found three major haplogroups, two of which were implicated in the global spread of the species [9]. Some of these haplogroups appeared more prevalent in particular climatic and geographic regions, such as haplogroup A1a which chiefly characterized the tropics and A1a2 which is mostly distributed in temperate regions. Another haplogroup (A2) appeared important in the spread of *Ae*. *albopictus* from SE Asia toward Australasia, distinguishing many of the samples from the Philippines, PNG, Indonesia and the Torres Strait Islands [9]. A similar pattern was observed using our *COI* data when viewing the nine haplotypes that account for 92% of the sequences in our study. Haplotype three (H3) was found to be common in Philippines, Indonesia, the Torres Strait Islands and Southern Fly Region but also present in historically established PNG populations, Vietnam and an individual from Sepilok, Borneo (Malaysia). Consequently, the populations from Vietnam, Borneo, the Philippines and Sulawesi could represent important unsampled populations that may influence ABC results and should be considered in future studies, which could additionally explain why the posterior probability of our most likely invasion scenario was moderate (P = 0.52). We also found that H1 was more prevalent in regions that experience temperate and subtropical climates, while H4 was widespread in the tropics (Fig 3). Due to the maternal inheritance of mtDNA, these results could suggest that the movement of females has been somewhat limited to their preadaptation to certain climatic regions. For example, females originating from a temperate region may be more being likely to successfully invade other temperate regions. It is possible that these genetic patterns for *COI* in *Ae*. *albopictus* are associated with the photoperiod response of different populations, which could contribute to higher gene flow and invasion success between climatically similar regions [17, 74–76]. However, the photoperiodic response of *Ae*. *albopictus* in recently introduced regions within Australasia is lacking (especially for New Guinea, the Solomon Islands, Fiji and Nauru). Within the Torres Strait Islands it appears the population is of tropical, Indonesian origin and egg survival was lower in less humid conditions [77], supporting this conclusion. However, it is worth noting that there are multiple other *COI* haplotypes that do not conform to any obvious geographic patterns (S6 Fig), suggesting that there have been multiple introduction events into some locations from mainland Asia or potentially unsampled locations—a similar pattern which was also highlighted by other studies [9, 74]. The possibility of insertion of mtDNA in the nuclear genome [78, 79] was considered in this study and our *COI* sequences were assessed by examining chromatograms (no double peaks in chromatograms) and by checking for stop codons. It seems unlikely that there is nuclear insertion of mtDNA in samples from our study—but it remains a possibility and requires further research.

We included several populations from the USA and Indian Ocean to explore how these populations fit into a broader geographic analysis with our samples, which are chiefly from SE Asia and Australasia. We did not include these in our ABC analysis because of the lack of temperate Asian populations, which have been shown as the source of USA introductions and because of insufficient sample sizes in Indian Ocean populations (Cocos (Keeling) Islands, Christmas Island and La Réunion). We included them in our other analyses to assist in future studies and have discussed them in S1 File, Supplementary Discussion.

### Invasion into Australasia

#### New Guinea

In New Guinea (PNG and Papua (Indonesia)), *Ae*. *albopictus* was first detected in Jayapura in the northeastern corner of Papua Province, Indonesia (formerly West New Guinea) in 1962 [55, 57]. By the early 1970s it was reported in northern PNG near Madang and was established in the PNG capital Port Moresby by the 1980s [55, 57, 58]. There were reports of *Ae*. *albopictus* from New Guinea earlier but these have been considered doubtful and probably referred to *Ae*. *scutellaris* [56]. However, we considered these earlier dates in our ABC prior distributions for testing the timing of introduction events. We found support for a later arrival of *Ae*. *albopictus* into Papua (~1959 [95% CI = 1943, 1967]), rather than earlier dates from the 1920s, but likewise, the introduction could have been much earlier than the 1962 detection [55]. Additionally, our ABC analysis show that the Papuan population of *Ae*. *albopictus* probably originated from mainland SE Asia, rather than from our sampled Indonesian population. The subsequent spread into PNG was supported as originating from a similar mainland SE Asian source in a separate and later event, rather than from an introduction from Indonesia (including both Papua and non-New Guinea Indonesian populations in our study). The timing of the introduction into PNG in the ABC analysis (~1970 [95% CI = 1963, 1976]) corresponded with first detection dates from 1972 [57], but similar to Papua, could have been earlier than first observed which is plausible given the lack of surveys in PNG at the time.

Previous data [18] from PNG used inconsistent M13 dyes for a given microsatellite locus, causing dye shifts [35] resulting in some incorrectly scored alleles. This was uncovered and corrected in the present study (as outlined in the Materials & Methods) and revealed less substructuring of PNG populations than previously suggested [18]. We found that all historically established PNG populations were a homogenous genetic cluster (whereas in the previous study, Kiunga and Port Moresby both appeared as distinct genetic clusters using microsatellites), but this dye shift had no discernible influence on the general relationships between the other populations in their study [18]. Cooper, Waterson [58] suggested that the dispersal of *Ae*. *albopictus* in PNG is primarily driven by aircraft and coastal shipping as there is not an extensive road network in PNG. Surprisingly, the small island of Daru, offshore of southern PNG, showed no detectable genetic change using bottleneck statistics between samples analysed after 16 years (1992–2008), nor did Port Moresby over 4 years (1996–1999); neutrality tests using *COI* revealed no contraction or expansion of these populations, but results were insignificant.

#### Solomon Islands, Fiji and Nauru

The introduction of *Ae*. *albopictus* into the Solomon Islands in 1979 and eventually into Fiji in 1989 was originally suspected to be from a progressive expansion, most likely from PNG via shipping [54, 80]. Our ABC analysis suggested the Solomon Islands introduction originated from either PNG or mainland SE Asia in ~1979 (95% CI = 1973, 1983), corresponding with the first detection date—due to moderate type I and II error it is unclear which region was the primary source of the introduction (see Results). Major commercial shipping routes exist between the Solomon Islands and mainland SE Asia and currently the Solomon Islands has significant trade with Singapore and Malaysia [81] which could explain the ABC results and high between-region gene flow (F_ST_ = 0.05–0.11). Large-scale logging operations have been prevalent in the Solomon Islands over the last 40 years, with a four-fold influx of logging industry multinationals, chiefly from SE Asia (especially Malaysia), during the early 1980s [82]. Shipping associated with this industry could have provided a prime opportunity for the introduction of *Ae*. *albopictus* from SE Asia. However, our STRUCTURE and DAPC plots also show the Solomon Islands has close genetic affinity to both mainland SE Asia and PNG clusters. Compared to SE Asia, PNG showed less, but relatively high gene flow with the Solomon Islands (F_ST_ = 0.08–0.20). Papua New Guinea has strong historical and contemporary cultural ties with the Solomon Islands and other islands of the Melanesian region (spanning the islands from PNG to Fiji) and human movements between the regions have been ongoing, which may explain PNG’s genetic ties to the Solomon Islands, particularly Lihir Island (S1 File, Supplementary Discussion: Lihir and Buka Islands). The majority of Solomon Islands *COI* sequences were H5, which is widespread throughout PNG; however, this haplotype is also observed in some mainland SE Asian populations (from Singapore and Thailand) as well as Indian Ocean Islands (Cocos (Keeling) Islands and Christmas Is.). Additionally, multiple private *COI* haplotypes were only found in the Solomon Islands, highlighting some unique structure in the Solomon Islands that was also shown at K = 9 in STRUCTURE (S1 Fig). For microsatellites we found there were no shared private alleles between the Solomon Islands and mainland SE Asia (i.e. alleles that were exclusively found in the two regions), but a single shared private allele was exclusive to the Solomon Islands and PNG. Overall, these results highlight that there have probably been multiple introductions into the Solomon Islands from both mainland SE Asia and PNG.

Due to insufficient sampling of Nauru and Fiji populations, we were unable to test these populations in our ABC analysis. Results are discussed in S1 File, Supplementary Discussion: Fiji and Nauru, but conclusions should be interpreted cautiously given small sample sizes. *Aedes albopictus* was only recently found on Nauru in April 2014 –first detected and morphologically identified by Michael Bangs, and genetically confirmed using PCR-restriction digest and *COI* in this study (see Materials and methods).

#### Torres Strait Islands and Southern Fly Region (Southern PNG)

In 1988, *Ae. albopictus* was detected in the coastal Southern Fly Region of PNG and on the nearby PNG island of Daru. However, a 1992 survey showed that while *Ae. albopictus* was still present on Daru, it was not found in/around the coastal villages of the Southern Fly Region. Consequently, Cooper, Waterson [58] highlighted that the species had failed to establish in these coastal locations after its initial introduction, despite an abundance of suitable larval habitats. However, a re-introduction occurred in 2005 at the same time as the Torres Strait incursion, which were suspected to be part of the same invasion wave into the region due to their genetic similarity [18]. Compared to Indonesian and historically established PNG populations, the Torres Strait/Fly Region invasive population was more genetically similar to Indonesian populations and was suspected to have been introduced by illegal, Indonesian fishing vessels [18]. Our ABC analysis supports the Indonesian origin of these introduced populations occurring ~2005 (95% CI = 2003, 2007). It is likely the Torres Strait and Fly Region were either seeded by a similar invasion source, or that local movements (mostly boat (no airstrips in the Fly Region)) after an initial invasion into the region have seeded new populations and homogenized population structure in the region. The presence of multiple shared *COI* haplotypes with PNG and mainland SE Asian populations also highlight that there have probably been multiple secondary introductions into the region, some potentially from unsampled populations. We found no support for initial founder effects after the Torres Strait/Fly Region introduction, but median N_e_ showed a gradual increase after its introduction, potentially suggesting growth of the population over time. Note that in our ABC analysis this region is represented as a random subset of multiple populations (due to the complexity of modelling each population separately), so these results are more reflective of the stabilization of N_e_ in the Torres Strait/Fly Region as a whole. In contrast, when we explored population structure on individual islands of the Torres Strait we found variable and sometimes drastic temporal changes in genetic structure (outlined below).

Our microsatellite results show high temporal genetic structure in the Torres Strait region over the past decade. Furthermore, our data indicate either a notable pattern of genetic drift on some islands (such as Keriri, Ngurupai and Warraber) or could represent a secondary introduction into the region from an unsampled population. We found evidence of genetic bottlenecks for several Torres Strait Islands, but only Waiben (2010 population) was supported by both bottleneck statistics used here (M-ratio and heterozygosity excess tests). Williamson-Natesan [83] highlighted that when a bottleneck is supported by the M-ratio, but not heterozygosity excess, it can indicate a more severe and older population bottleneck and vice versa for weaker and more recent bottleneck events. Consequently, we found evidence for older and more severe bottlenecks on Mabuiag, Waiben, Ngurupai from earlier collections after the initial invasion of *Ae*. *albopictus* into the region, as well as in more recent collections from Poruma and Iama. The dramatic wet-dry climate in the Torres Strait [77, 84], intense spraying efforts and refocusing of control efforts (see S1 File, Supplementary Discussion: Control and surveying on the Torres Strait Islands; and [27, 28]) could explain population bottlenecks on certain islands and the differing genetic trajectories of the islands. Additionally, initial founder effects (resulting from small invading populations) could have played a strong role in the pattern of genetic drift on the various islands. Laboratory experiments by Nicholson, Ritchie [77] showed that hotter, dryer conditions significantly reduced egg viability in *Ae*. *albopictus* from Masig Is. in the Torres Strait, so it is possible that seasonal changes have also caused genetic bottlenecks, but it is likely there are a multitude of factors at play. Both bottleneck tests used here have been shown to fail to detect recent bottlenecks (within 1–5 generations), particularly given small sample sizes and few loci [49, 85]–to more accurately determine if a recent bottleneck has occurred more extensive temporal sampling will need to be conducted in the future. As previously mentioned, it remains a possibility that a secondary introduction from an unsampled source has caused the rapid temporal genetic changes observed on some islands. Indeed, Urbanelli, Bellini [67] showed that the Sulawesi region harbours highly distinct populations that can be distinguished from other Indonesian populations of *Ae*. *albopictus* and the region could be key to fully understanding the species’ invasion of Australasia. Likewise, Southern PNG is highly connected to the Torres Strait Islands via local boating traffic [28] and the temporal genetic change detected could have originated from an unsampled population in this region.

### Conclusion and future implications

For the first time, we have used ABC analysis to compare various invasion scenarios of *Ae*. *albopictus* in the Australasian region and have additionally explored and characterised the genetic structure of a wide range of populations in the Indo-Pacific, which had not been compared under a single study. We uncovered notable temporal population structure in recently introduced Torres Strait Island populations. Importantly, this demonstrates some of the drastic changes that invading populations may undergo within a short time period (i.e. in less than a decade), which has substantial implications on the practicality and accuracy of using such genetic databases for estimating invasion sources. However, we also found that historically established populations of *Ae*. *albopictus* displayed stable population structure. Future studies that aim to address the global genetic structure of *Ae*. *albopictus* will need to consider the full native range of the species and the influence of temporal collections on population structure, especially newly established populations. Likewise, ABC analyses that account for complex scenarios (requiring thorough spatio-temporal sampling) and gene flow between populations will play a key role in better understanding the population dynamics of *Ae*. *albopictus* as well as other mosquito species that are highly associated with humans, such as *Ae*. *aegypti*. The standardisation of genotyping methods and sampling efforts will allow for more rigorous assessments of the global population structure of *Ae*. *albopictus*, given the scale of its current distribution which makes such population studies logistically challenging. This will prove essential in controlling the spread of *Ae*. *albopictus* and for assessing the health risk of different populations, given their variation in vector efficiency, physiology and behaviour [10, 86–88].

## Supporting information

S1 Table Specific sample information for *Aedes albopictus* used in the microsatellite study for 911 individual mosquitoes, including microsatellite allele scores for 13 loci. LC, larval collection; IC, immature (pupal or larval) collection; EC, egg collection; HLC, human landing collection; HBS, human baited sweep netting; LT, light trap; ST, sentinel trap; ASP, battery-powered aspirator; unknown details are shown (-).
(XLSX)

S2 Table Matrices of pairwise F_ST_ (A), G"_ST_ (B) and Jost's D (C) values for populations of *Aedes albopictus* using 13 microsatellite loci. P values are indicated above the diagonal with insignificant (P>0.05) in bold.
(XLSX)

S3 Table Details used for approximate Bayesian computation (ABC) analysis for investigating the Australasian invasion of *Aedes albopictus*. Included are various parameter settings and conditions used in our ABC analysis and the posterior distributions of our most likely scenarios (Scenario 4; Scenarios 1 & 4). Results for the confidence in scenario choice are also displayed (type I and type II error).
(XLSX)

S4 Table Sample information for mtDNA *COI* sequences, representing 1044 samples of *Ae*. *albopictus*. (XLSX)

S5 Table Estimates of genetic diversity using all 13 microsatellite loci within populations of *Aedes albopictus*. Displayed are the mean values per population and the standard error ([SE]): mean population size (N), mean number of alleles (Na), number of effective alleles (Ne), observed heterozygosity (Ho), expected heterozygosity (He), unbiased expected heterozygosity (uHe) and fixation index (F).
(XLSX)

S6 Table Summary of mtDNA *COI* haplotype distribution across 97 populations of *Ae*. *albopictus*, containing a total of 1044 individual sequences and 52 unique haplotypes. (XLSX)

S7 Table Tajima’s D and significance value (P value < 0.05) as well as nucleotide diversity (π) using *COI* sequences for populations of *Aedes albopictus* that had temporal collections from the present study. Sample sizes varied by year and are indicated. We found no significance (P > 0.05) in all calculations of Tajima’s D.
(XLSX)

S8 Table Garza-Williamson index (M-ratio) and Wilcoxon test for heterozygosity excess (P value) for genetic bottlenecks in populations of *Aedes albopictus* using 13 microsatellite loci. Values in bold indicate a bottleneck (M-ratio ≤ 0.68; two-tailed Wilcoxon test P < 0.05). Populations from the Southern Fly Region, Nauru and Port Moresby (1996, 1997) were not included due to insufficient data (see Table 1 for n).
(XLSX)

S1 Fig Bayesian STRUCTURE plot (K = 9) for 13 microsatellite loci for 911 samples of *Aedes albopictus* in the study region. Each vertical bar in the plots represents an individual sample, where the color of the bar indicates the probability of the individual belonging to a genetic cluster. Samples are positioned on the map corresponding to the population’s location (orange dot) and are abbreviated as in Table 1. Map insets represent the following: **A)** Torres Strait Islands and Southern Fly Region of Papua New Guinea; **B)** Hawaii; **C)** Atlanta. Insets B and C are to scale with the main map scale. The top-left color key shows the color of clusters, as referred to in the main text.
(TIF)

S2 Fig **A) DAPC of the full dataset for 13 microsatellite loci for *Aedes albopictus* in the study region.** Three-dimensional plots show the relationship between individuals belonging to 23 different populations (represented by colored dots, where the color of a dot corresponds to population) using the first 3 principal components (PC1-3). Each plot shows the same data, but is rotated along the horizontal plane. Four distinct clusters (C1-4) are indicated with dashed ellipses (not confidence intervals) and cluster membership of each population is denoted in the legend. This plot is chiefly to visualize the genetic relationships between the four main clusters and specific relationships are discussed in text. **B)** Correspondence analysis (CA) of the same data, but presenting population means rather than individual data points. Note that for both plots A and B, population definitions varied slightly from STRUCTURE analyses and are shown in Table 1 along with population abbreviations.
(TIF)

S3 Fig DAPC analysis for 13 microsatellite loci for *Aedes albopictus* in the study region. **A-B)** Scatterplots show the front (**A**) and top (**B**) view of a DAPC for the reduced dataset of *Ae*. *albopictus* (n = 458, containing only C3 and C4 from S2A Fig), using the first 3 principal components (PC1-3). This excludes populations from the Torres Strait Islands, Jakarta, Sumba, Timor-Leste and Southern Fly Region. Individuals from each of the 18 populations are color coded and labeled with a number (see legend in plot A). Note that Solomon Islands includes Gizo, New Mala and Honiara and that Singapore and Malaysia are treated as one population. Ellipses show the 95% confidence intervals of each population.
(TIF)

S4 Fig Principal components analysis (PCA) in the space of summary statistics computed for our DIYABC simulations (across all scenarios). Each colored dot represents a simulated dataset corresponding to the five scenarios (with 10,000 random prior plots displayed per scenario), while the large yellow dot represents our observed dataset. The first three principal components (PC) are shown with their % variance explained by each PC shown in brackets.
(TIF)

S5 Fig Principal components analysis (PCA) in the space of summary statistics used for the most likely Australasian invasion scenario (Scenario 4) in our study. The yellow dot represents the observed *Ae*. *albopictus* dataset, solid purple dots represent the simulated dataset with parameters drawn from posterior distributions (1,000 random datasets shown), while hollow purple dots corresponds to the datasets simulated based on prior distributions of parameters (1,000 random datasets shown). The % variance explained by principal components (PC) is displayed in brackets and only the first three PCs are plotted.
(TIF)

S6 Fig Mitochondrial *COI* haplotype network for 52 haplotypes identified for 1044 individuals of *Aedes albopictus* in the Indo-Pacific, Asian and USA region. Haplotypes are colored by broad geographic region and the size of circles indicates the number of individuals belonging to a given haplotype. Lines joining haplotypes show genetic distance between haplotypes where each mark indicates a single nucleotide substitution. Small black circles represent unsampled haplotypes.
(TIF)

S1 File Supplementary methods, results and discussion section. (DOCX)
