# Supplementary figures and images for: Tiger on the prowl: Invasion history and spatio-temporal genetic structure of the Asian tiger mosquito Aedes albopictus (Skuse 1894) in the Indo-Pacific
Source: PLoS Negl Trop Dis. 2017 Apr 14;11(4):e0005546. doi: 10.1371/journal.pntd.0005546 (PMC5406021; doi:10.1371/journal.pntd.0005546)

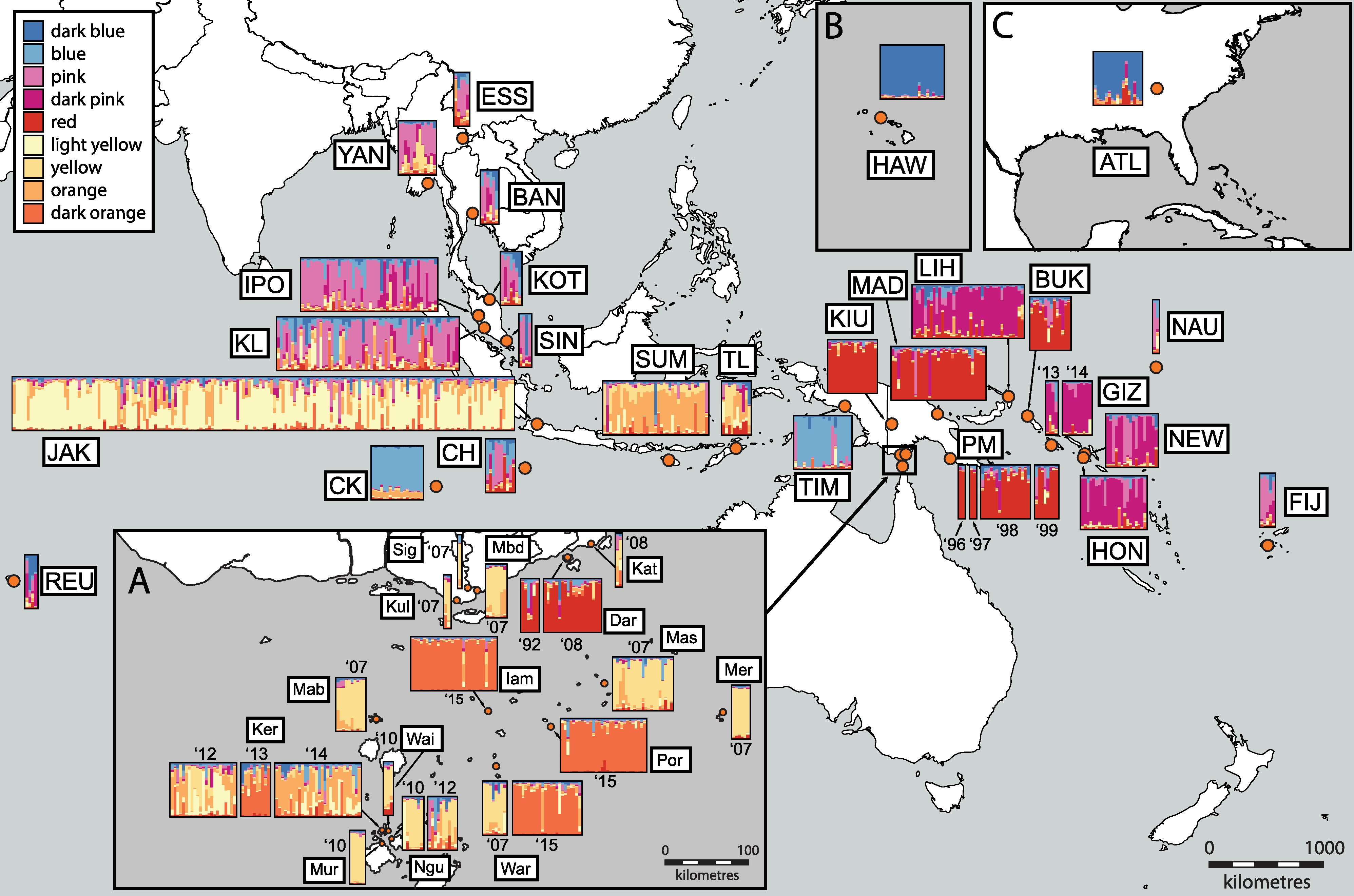

Supplement: S1 Fig — Each vertical bar in the plots represents an individual sample, where the color of the bar indicates the probability of the individual belonging to a genetic cluster. Samples are positioned on the map corresponding to the population’s location (orange dot) and are abbreviated as in Table 1. Map insets represent the following: A) Torres Strait Islands and Southern Fly Region of Papua New Guinea; B) Hawaii; C) Atlanta. Insets B and C are to scale with the main map scale. The top-left color key shows the color of clusters, as referred to in the main text. (TIF) [file pntd.0005546.s009.tif]

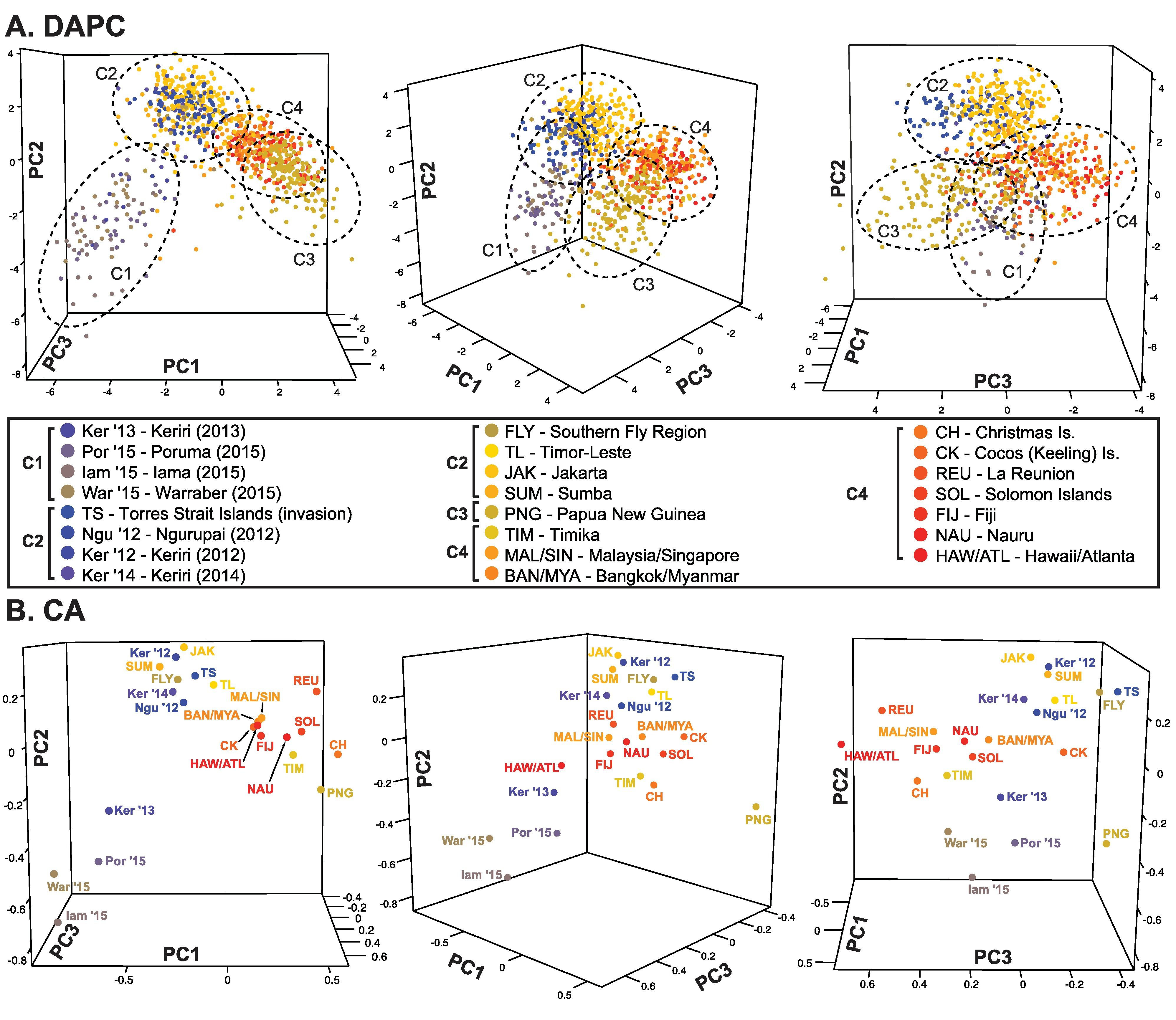

Supplement: S2 Fig — A) DAPC of the full dataset for 13 microsatellite loci for Aedes albopictus in the study region. Three-dimensional plots show the relationship between individuals belonging to 23 different populations (represented by colored dots, where the color of a dot corresponds to population) using the first 3 principal components (PC1-3). Each plot shows the same data, but is rotated along the horizontal plane. Four distinct clusters (C1-4) are indicated with dashed ellipses (not confidence intervals) and cluster membership of each population is denoted in the legend. This plot is chiefly to visualize the genetic relationships between the four main clusters and specific relationships are discussed in text. B) Correspondence analysis (CA) of the same data, but presenting population means rather than individual data points. Note that for both plots A and B, population definitions varied slightly from STRUCTURE analyses and are shown in Table 1 along with population abbreviations. (TIF) [file pntd.0005546.s010.tif]

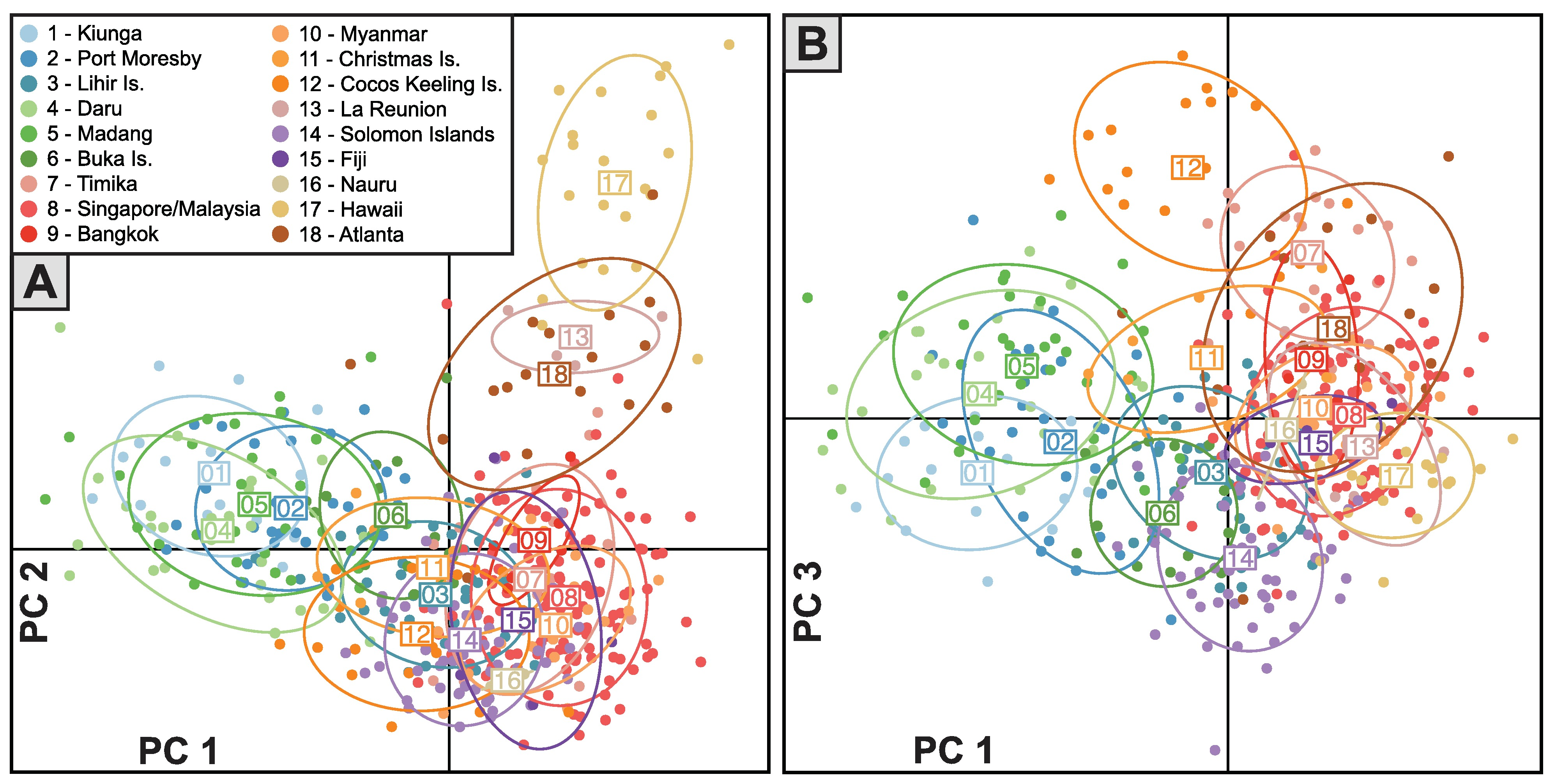

Supplement: S3 Fig — A-B) Scatterplots show the front (A) and top (B) view of a DAPC for the reduced dataset of Ae. albopictus (n = 458, containing only C3 and C4 from S2A Fig), using the first 3 principal components (PC1-3). This excludes populations from the Torres Strait Islands, Jakarta, Sumba, Timor-Leste and Southern Fly Region. Individuals from each of the 18 populations are color coded and labeled with a number (see legend in plot A). Note that Solomon Islands includes Gizo, New Mala and Honiara and that Singapore and Malaysia are treated as one population. Ellipses show the 95% confidence intervals of each population. (TIF) [file pntd.0005546.s011.tif]

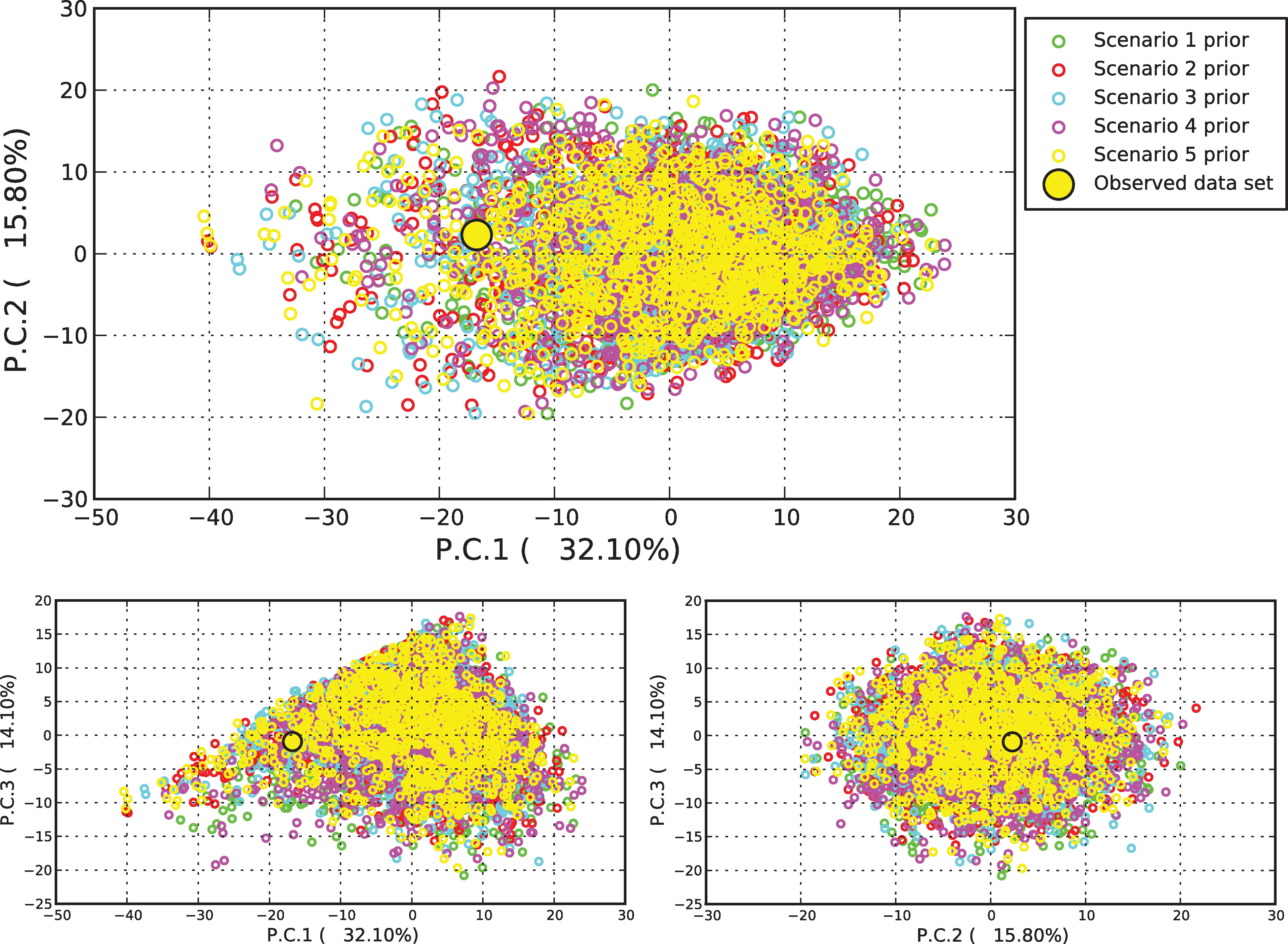

Supplement: S4 Fig — Each colored dot represents a simulated dataset corresponding to the five scenarios (with 10,000 random prior plots displayed per scenario), while the large yellow dot represents our observed dataset. The first three principal components (PC) are shown with their % variance explained by each PC shown in brackets. (TIF) [file pntd.0005546.s012.tif]

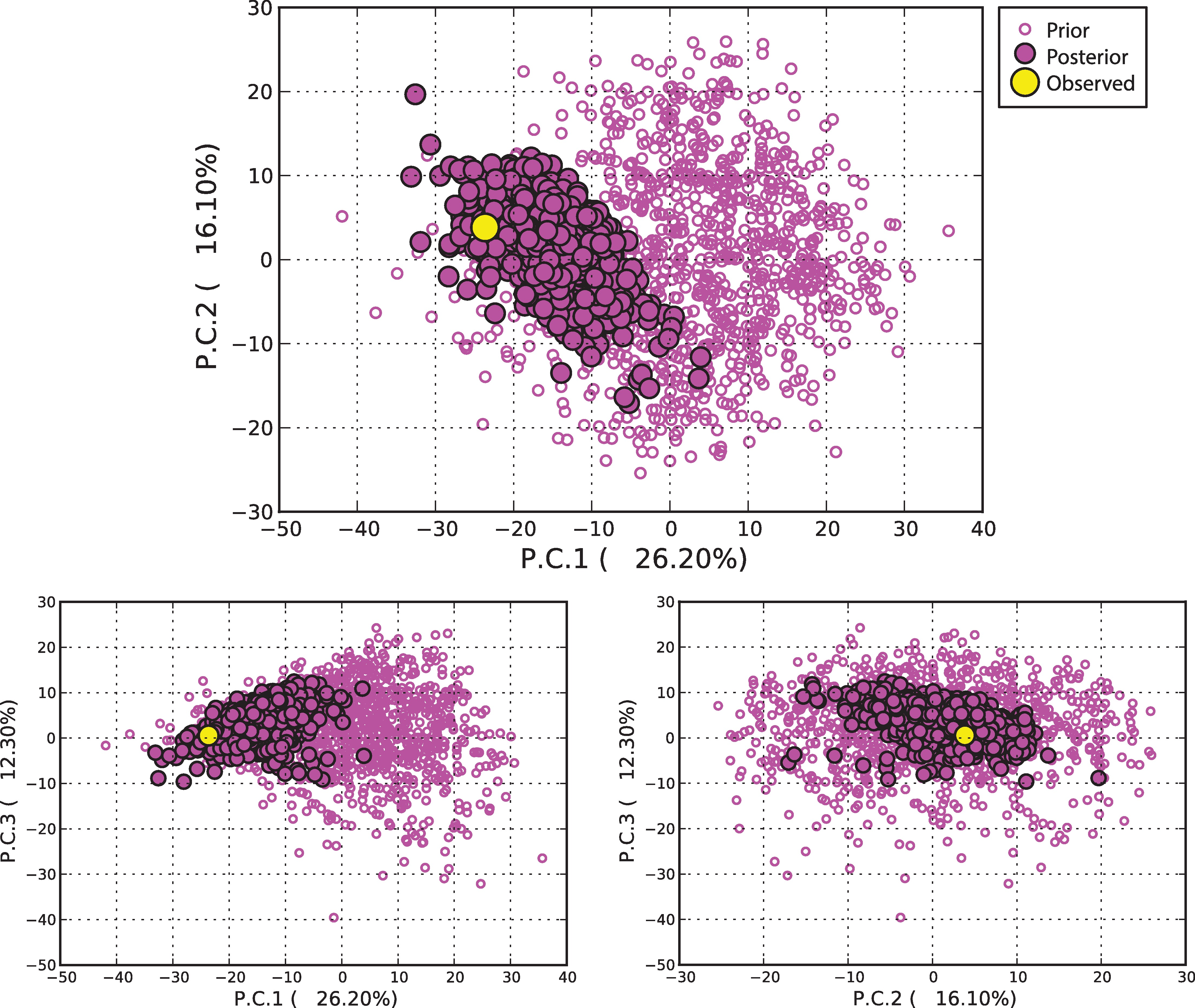

Supplement: S5 Fig — The yellow dot represents the observed Ae. albopictus dataset, solid purple dots represent the simulated dataset with parameters drawn from posterior distributions (1,000 random datasets shown), while hollow purple dots corresponds to the datasets simulated based on prior distributions of parameters (1,000 random datasets shown). The % variance explained by principal components (PC) is displayed in brackets and only the first three PCs are plotted. (TIF) [file pntd.0005546.s013.tif]

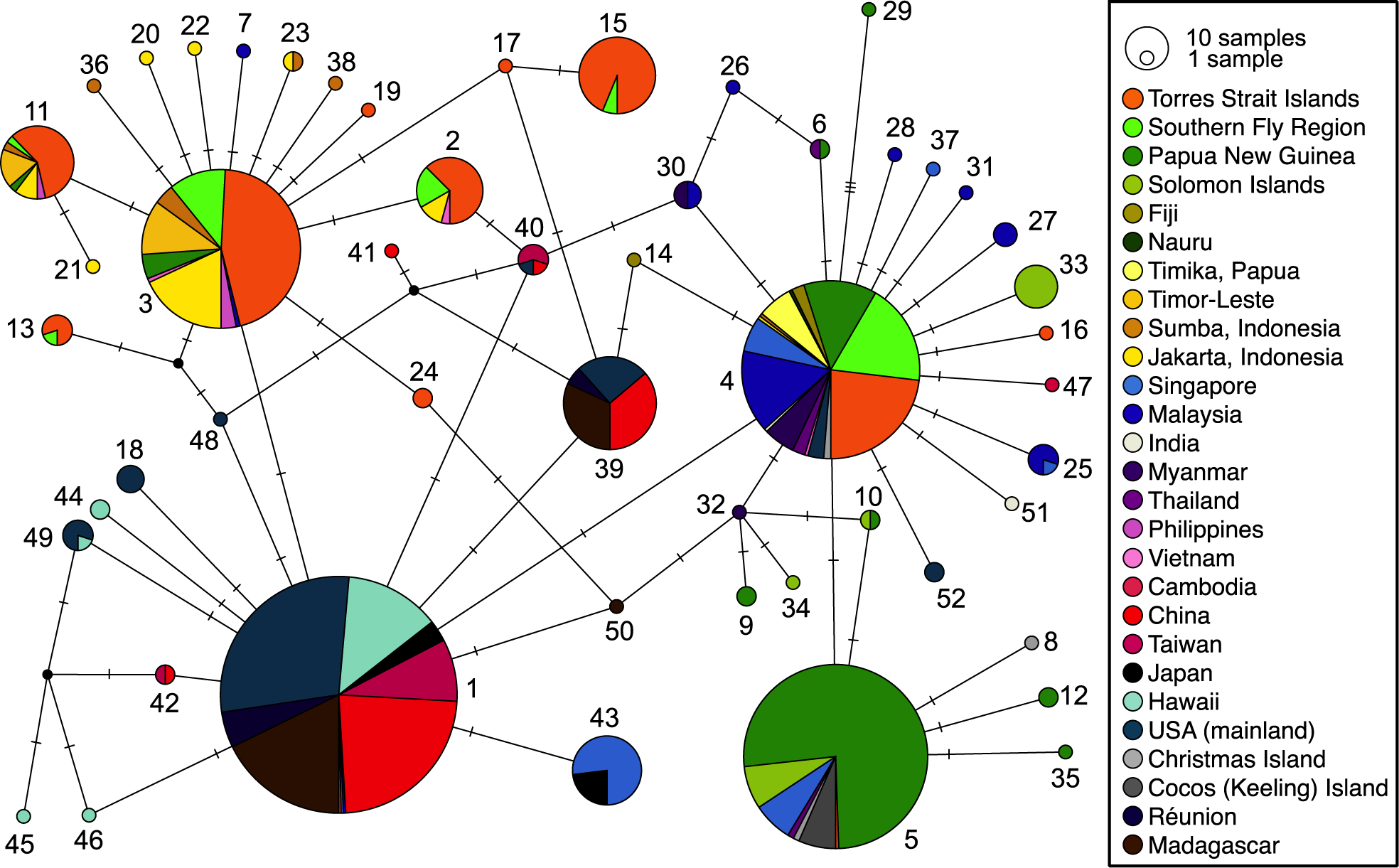

Supplement: S6 Fig — Haplotypes are colored by broad geographic region and the size of circles indicates the number of individuals belonging to a given haplotype. Lines joining haplotypes show genetic distance between haplotypes where each mark indicates a single nucleotide substitution. Small black circles represent unsampled haplotypes. (TIF) [file pntd.0005546.s014.tif]
