# Supplementary material for: Tiger on the prowl: Invasion history and spatio-temporal genetic structure of the Asian tiger mosquito Aedes albopictus (Skuse 1894) in the Indo-Pacific
Source: PLoS Negl Trop Dis. 2017 Apr 14;11(4):e0005546. doi: 10.1371/journal.pntd.0005546 (PMC5406021; doi:10.1371/journal.pntd.0005546)
Supplement: S1 File — (DOCX) [file pntd.0005546.s015.docx]

**File S1 – Supplementary File**

**SUPPLEMENTARY METHODS**

**Preliminary STRUCTURE**

Preliminary analyses were run with K ranging from 2 – 20 (5 iterations per value of K) for 200,000 generations per iteration with a burn-in of 50,000. These analyses were run separately with and without location priors to assess its effect on clustering; final analyses were run with these priors as we found no major effect on clustering. Analyses were run with the admixture model to allow for mixed ancestry in individuals [1]. The preliminary STRUCTURE output was run in STRUCTUREHARVESTER [2] to assess the likelihood values across K and to infer the most likely value of K (using the Evanno ∆K method [3] and also by assessing L (K)). Additionally, we considered the value of K based on what we perceived to be the most efficient and biologically relevant summary of the data, as realistically a true K is difficult to determine.

**Performance of DIYABC**

A PCA was performed to check congruency between the observed dataset and the distribution of summary statistics based on priors (Fig S4); one should expect the observed data to fall within the PCA data cloud for priors. We used the logistic regression approach [4] to estimate the posterior probability of our tested scenarios using 1% of the simulated datasets closest to the observed data. Confidence in scenario choice was assessed by estimating type I and type II errors using 500 pseudo-observed datasets drawn from prior distributions of a given scenario [5]. The posterior distributions of parameters were estimated for the most likely scenario (Scenario 4) using 1% of the simulated data closest to the observed, and we computed bias and precision of these parameter estimations using 500 pseudo-observed datasets drawn from the posterior distributions. Model checking was also conducted on the most likely scenario (Scenario 4) using PCA in the space of all one and two sample summary statistics using 1000 datasets simulated from the posterior distributions of parameters. If the model fits our data well, both prior and posterior simulated datasets will show overlap with the observed dataset on the PCA planes (Fig S5) [5].

**SUPPLEMENTARY RESULTS**

**STRUCTURE (K=9)**

At K=9, mainland SE Asia (pink), USA (dark blue) and the Solomon Islands (dark pink) split into more distinct clusters. Christmas Is. appears more similar to Timika and mainland SE Asia, whereas both Cocos (Keeling) Islands and La Réunion appears most similar to populations from SE Asia (however, the sample size for La Réunion is small (n = 4)). The Indonesian (light green) cluster present at K=4 (Fig 1) contains substructure which is visible at K=9 and separates Jakarta from Sumba, Timor-Leste and Torres Strait Islands and Fly Region populations (Fig S1). At K=9, the Jakarta population (light yellow) appears distinct from a group containing populations from Timor-Leste and Sumba (orange) and another cluster mostly containing the PNG Southern Fly Region and Torres Strait Islands (collections between 2006 – 2012) (yellow). Similar to the lower K value results, the more recent collections from the Torres Strait Islands (2013 – 2015) form a distinct cluster (dark orange) from the older collections (yellow). A subset of Torres Strait Island populations appear more similar to the Sumba/Timor-Leste cluster (orange) (see Ker ‘12, Ker ‘14, Ngu ’12) but also contain admixture with the Jakarta cluster (light yellow) as well as Timika/Cocos (Keeling) Islands (blue). The historically-established PNG populations, (excluding Lihir Is.), remain distinct as their own private cluster (red), although some individuals show signs of admixture with the Solomon Islands and mainland SE Asia (pink and dark pink). At K=9, Lihir Is. clusters with the Solomon Islands (dark pink) whereas at K=4 most individuals clustered with PNG**.** In addition, Timika forms a separate cluster with similar structure to the Cocos (Keeling) Island population (blue); both of these populations also have a notable degree of admixture with mainland SE Asia.

**SUPPLEMENTARY DISCUSSION**
**USA**

As with many previous studies [6-11], we found that the United States (mainland USA and Hawaii) populations of *Ae. albopictus* appear more closely related to temperate/northern Asian populations than to more southern, tropical SE Asian populations, as evidenced by their similarity in *COI* haplotypes. One of the limitations of our study is that it does not include microsatellite samples from temperate Asian populations (e.g. China, Korea and Japan). There are however, multiple shared haplotypes between the USA and mainland SE Asian populations, which could reflect both historic and contemporary movements between these regions. Additionally, microsatellite genetic distance measures show that USA populations are most similar to mainland SE Asian populations (F_ST_ = 0.128 - 0.23) within our study, but are still quite distant. Despite the lack of temperate Asian samples in our microsatellite analyses, we show that at K=4 populations from the USA appear in the same genetic cluster as mainland SE Asia, while at K=9 they form their own distinct cluster.

**Indian Ocean Islands**

*Madagascar and La Réunion*

Populations of *Ae. albopictus* from Madagascar and La Rèunion are largely thought to have originated from SE Asia as a result of human movements, particularly due to the multiple dispersal opportunities associated with human migration waves (as early as 1,500 - 2,000 ybp based on anthropological data [12] and the spice trade occurred (during the 17^th^ and 18^th^ centauries). Interestingly, we found that Madagascar and La Réunion share most *COI* haplotypes with the USA and subtropical and temperate regions of Asia (China, Taiwan, Japan). Delatte, Bagny (12) showed that there are multiple distinct genetic groups in Madagascar and La Rèunion that correspond to an ancient lineage and a more widespread contemporary lineage. More recently, Manni, Guglielmino (13) found some support that the introduction into La Rèunion was of SE Asian origin (genetically similar to a Thailand population of *Ae. albopictus*), but due to overlapping 95% confidence intervals, this could have alternatively been a derivation from an admixture event (between Thailand and Japan) or from China. In addition, the species has recently (since the 1980s) undergone a significant expansion in its distribution in the eco-climatically diverse region of Madagascar [14], where the species’ ecological plasticity (along with changes in rain regimes and human movements) has facilitated its establishment in coastal, tropical and high altitude, temperate habitats. Potential admixture with (and colonisation of) lineages from temperate Asia and the USA may also explain the species’ recent range expansion in Madagascar and ability to exploit temperate conditions. China represents a key import source for Madagascar (20.6% of inter-country trade) and this could explain shared *COI* haplotypes between these regions [15]. Indeed, it is apparent that COI haplotypes may be associated with the overwintering eggs of *Ae. albopictus*, with H4 being more prevalent in regions that experience temperate climate.

*Cocos (Keeling) Islands and Christmas Island*

The Cocos (Keeling) Islands and Christmas Island populations are thought to have been introduced in two separate events, both of suspected mainland SE Asian or Indonesian origin (refs). The Cocos (Keeling) Islands introduction represents a more historical invasion (probably introduced between 1879 – 1905 [16]) whereas Christmas Island was colonised by *Ae. albopictus* in the early 1990s (not detected in 1989, but found after 1996 [17]). Our microsatellite analyses show that both populations are genetically distinct from each other which could reflect their isolation and different introduction sources. Microsatellites revealed that both islands are genetically similar to mainland SE Asian populations (excluding Myanmar: F_ST_ = 0.071 - 0.121), supporting their suspected introduction source and corresponding to historical routes taken by British and Dutch vessels for Cocos (Keeling) Islands and more contemporary movements for Christmas Island [16, 17]. Interestingly, *COI* haplotypes found on the islands (H4 & H5) have a wide distribution in the Indo-Pacific and do not assist in discerning the origin/s of the populations. Note that our sample sizes were small and results are not overly convincing of the introduction source of *Ae. albopictus* on the islands and further investigation is needed.

**Lihir and Buka Islands (PNG)**

Geographically, Lihir Is. and Buka Is. are part of the northern Solomon archipelago, but administratively part of PNG. Interestingly, the Lihir Is. population clusters mostly with the PNG cluster when K=4, but more so with the Solomon Island populations when K=9. In contrast, Buka Is. clusters consistently with the mainland PNG cluster at both values of K. The most likely explanation for this pattern could be that human connectivity, influenced by geo-political boundaries and commercial activity between the Solomon Islands and PNG, may be influencing the population structure within the PNG-Solomon region. Studies on the malaria mosquitoes (*Anopheles*) throughout the same region reveal strikingly different population genetic relationships [18, 19], but the dispersal of these species are not as closely associated with humans as *Ae. albopictus*. However, we cannot reject the idea of temporal variation contributing to the observed genetic structure in these populations, generated by the timing of collections – Buka Is. collections were from 1999 compared to Solomon Is. in 2013-2014 and Lihir Is. in 2007.

**Fiji and Nauru**

Our results showed that Fiji and Nauru were more similar to mainland SE Asia (F_ST_ = 0.024 - 0.116) than to the Solomon Islands (F_ST_ = 0.039 - 0.118), but more samples from Fiji and Nauru are needed to clarify this relationship. Fiji has significant import trade with Singapore and Nauru imports primarily from Fiji [20]. In addition, there are weekly flights between Nauru and Fiji. Clear population structure has been recently shown for *Ae. aegypti* in the South Pacific, which researchers suspected could be driven by a combination of factors including human-mediated movements, island isolation and environmental factors [21]. *Aedes aegypti* has been established since the early 19^th^ century in the region, so it is likely that the population dynamics of the more recently introduced *Ae. albopictus* differs, but the two species probably share similar modes of human-mediated dispersal. It is suspected that Fiji was the major source of introductions of *Ae. albopictus* into nearby regions such as Tonga and Vanuatu through intense sea and air traffic [21, 22], but this is yet to be tested under a coalescent framework (such as ABC analysis) and the chance of long distance introduction events from outside the South Pacific can not be ruled out at this point.

**Control and surveying on the Torres Strait Islands**

More than a decade after its first detection in the Torres Strait, *Ae. albopictus* is currently well established in large numbers and dominates the mosquito fauna on many Torres Strait islands (especially outer coral cay islands such as Masig, Poruma, Erub and Warraber) [23]. Most of the outer Torres Strait islands (furthest from Australia’s northernmost mainland tip – Cape York) have had *Ae. albopictus* since its initial detection in 2005. A control program attempted to eradicate *Ae. albopictus* from all outer islands between 2006-2008, but from 2009 onwards control efforts were shifted to the inner islands (those closer to the Australian mainland with direct transport nodes) Ngurupai and Waiben, along with the establishment of stricter quarantine zones in this inner region to prevent establishment on mainland Australia. Between 2006 and 2010, adult collections (human-baited sweep netting) on Masig, Poruma and Warraber islands revealed evidence of declines in the population sizes of *Ae. albopictus* in the dry season. However, after control efforts refocused on inner islands post 2009, there was substantial growth in population sizes by 2010 and it is assumed that populations have continued growing due to the lack of control measures [24]. Few surveys have been conducted on these outer islands since 2010, but a recent survey in 2016 found *Ae. albopictus* in high densities on many outer islands [24]. These surveys were conducted due to an outbreak of dengue on the outer islands of Darnley and Badu in early 2016, supposedly linked to a large outbreak on Daru and other regions of Western Province, PNG [24].

**REFERENCES**

1. Pritchard JK, Wen X, Falush D. Documentation for structure software: Version 2.3. 2009.

2. Earl D, vonHoldt B. STRUCTURE HARVESTER: a website and program for visualizing STRUCTURE output and implementing the Evanno method. Conservation Genet Resour. 2012;4(2):359-61. doi: 10.1007/s12686-011-9548-7.

3. Evanno G, Regnaut S, Goudet J. Detecting the number of clusters of individuals using the software STRUCTURE: a simulation study. Molecular Ecology. 2005;14(8):2611-20. Epub 2005/06/23. doi: 10.1111/j.1365-294X.2005.02553.x. PubMed PMID: 15969739.

4. Cornuet J-M, Pudlo P, Veyssier J, Dehne-Garcia A, Gautier M, Leblois R, et al. DIYABC v2. 0: a software to make approximate Bayesian computation inferences about population history using single nucleotide polymorphism, DNA sequence and microsatellite data. Bioinformatics. 2014;30(8):1187-9.

5. Cornuet JM, Pudlo P, Veyssier J, Loire E, Santos F, Dehne-Garcia A, et al. DIYABC version 2.1 - A user-friendly software for inferring population history through Approximate Bayesian Computations using microsatellite, DNA sequence and SNP data. 2015.

6. Birungi J, Munstermann LE. Genetic structure of *Aedes albopictus* (Diptera: Culicidae) populations based on mitochondrial ND5 sequences: evidence for an independent invasion into Brazil and United States. Annals of the Entomological Society of America. 2002;95. doi: 10.1603/0013-8746(2002)095[0125:gsoaad]2.0.co;2.

7. Kambhampati S, Black IV WC, Rai KS. Geographic origin of the US and Brazilian *Aedes albopictus* inferred from allozyme analysis. Heredity. 1991;67(1):85-94.

8. Urbanelli S, Bellini R, Carrieri M, Sallicandro P, Celli G. Population structure of *Aedes albopictus* (Skuse): the mosquito which is colonizing Mediterranean countries. Heredity. 2000;84(3):331-7.

9. Zhong D, Lo E, Hu R, Metzger ME, Cummings R, Bonizzoni M, et al. Genetic analysis of invasive *Aedes* *albopictus* populations in Los Angeles County, California and its potential public health impact. PLoS ONE. 2013;8(7):e68586. Epub 2013/07/19. doi: 10.1371/journal.pone.0068586. PubMed PMID: 23861921; PubMed Central PMCID: PMC3702605.

10. Morales Vargas RE, Phumala-Morales N, Tsunoda T, Apiwathnasorn C, Dujardin J-P. The phenetic structure of *Aedes albopictus*. Infection, Genetics and Evolution. 2013;13:242-51. doi: http://dx.doi.org/10.1016/j.meegid.2012.08.008.

11. Mousson L, Dauga C, Garrigues T, Schaffner F, Vazeille M, Failloux AB. Phylogeography of *Aedes* (*Stegomyia*) *aegypti* (L.) and *Aedes* (*Stegomyia*) *albopictus* (Skuse) (Diptera: Culicidae) based on mitochondrial DNA variations. Genetics Research. 2005;86(1):1-11. Epub 2005/09/27. doi: 10.1017/s0016672305007627. PubMed PMID: 16181519.

12. Delatte H, Bagny L, Brengue C, Bouetard A, Paupy C, Fontenille D. The invaders: phylogeography of dengue and chikungunya viruses *Aedes* vectors, on the South West islands of the Indian Ocean. Infection, Genetics and Evolution. 2011;11(7):1769-81.

13. Manni M, Guglielmino CR, Scolari F, Vega-Rúa A, Failloux A-B, Somboon P, et al. Genetic evidence for a worldwide chaotic dispersion pattern of the arbovirus vector, *Aedes albopictus*. PLoS Neglected Tropical Diseases. 2017;11(1):e0005332.

14. Raharimalala F, Ravaomanarivo L, Ravelonandro P, Rafarasoa L, Zouache K, Tran-Van V, et al. Biogeography of the two major arbovirus mosquito vectors, *Aedes aegypti* and *Aedes albopictus* (Diptera, Culicidae), in Madagascar. Parasites & Vectors. 2012;5(1):56. PubMed PMID: doi:10.1186/1756-3305-5-56.

15. DFAT. Madagascar: Department of Foreign Affairs and Trade; 2016 [13 May 2016]. Available from: https://dfat.gov.au/trade/resources/Documents/masy.pdf.

16. Murray M, Marks E. Blood-sucking Diptera of the Cocos (Keeling) Islands. Austral Entomology. 1984;23(4):265-8.

17. Harrington S, Lindsay M, Douglas A. Christmas Island and Cocos (Keeling) Islands, Indian Ocean: Mosquito fauna and mosquito-borne disease risk assessment and management recommendations. Western Australia Department of Health, 2009.

18. Ambrose L, Cooper RD, Russell TL, Burkot TR, Lobo NF, Collins FH, et al. Microsatellite and mitochondrial markers reveal strong gene flow barriers for *Anopheles farauti* in the Solomon Archipelago: implications for malaria vector control. International Journal for Parasitology. 2014;44(3-4):225-33. Epub 2014/01/21. doi: 10.1016/j.ijpara.2013.12.001. PubMed PMID: 24440418.

19. Seah IM, Ambrose L, Cooper RD, Beebe NW. Multilocus population genetic analysis of the Southwest Pacific malaria vector *Anopheles punctulatus*. International Journal for Parasitology. 2013;43(10):825-35. doi: http://dx.doi.org/10.1016/j.ijpara.2013.05.004.

20. DFAT. Nauru: Department of Foreign Affairs and Trade; 2016 [13 May 2016]. Available from: http://dfat.gov.au/trade/resources/Documents/naur.pdf.

21. Calvez E, Guillaumot L, Millet L, Marie J, Bossin H, Rama V, et al. Genetic diversity and phylogeny of *Aedes aegypti*, the main arbovirus vector in the Pacific. PLoS Neglected Tropical Diseases. 2016;10(1):e0004374-e.

22. Guillaumot L, Ofanoa R, Swillen L, Singh N, Bossin HC, Schaffner F. Distribution of *Aedes albopictus* (Diptera, Culicidae) in southwestern Pacific countries, with a first report from the Kingdom of Tonga. Parasites & Vectors. 2012;5(1):247.

23. van den Hurk AF, Nicholson J, Beebe NW, Davis J, Muzari OM, Russell RC, et al. Ten years of the tiger: *Aedes albopictus* presence in Australia since its discovery in the Torres Strait in 2005. One Health. 2016;2:19-24.

24. Muzari MO, Devine G, Davis J, Crunkhorn B, Van Den Hurk A, Whelan P, et al. Holding back the tiger: successful control program protects Australia from *Aedes albopictus* expansion. PLoS Neglected Tropical Diseases. 2017;11(2):e0005286.
